# “Personally, I feel sorry, but professionally, I don't have a choice.”^1^ Understanding the drivers of anti-Roma discrimination on the rental housing market

**DOI:** 10.3389/fsoc.2023.1223205

**Published:** 2023-07-18

**Authors:** Luca Váradi, Blanka Szilasi, Anna Kende, Jeremy Braverman, Gábor Simonovits, Bori Simonovits

**Affiliations:** ^1^Nationalism Studies Program, Central European University, Vienna, Austria; ^2^Institute of Psychology, ELTE Eötvös Loránd University, Budapest, Hungary; ^3^Media Hub, Central European University, Vienna, Austria; ^4^Department of Political Science, Central European University, Vienna, Austria; ^5^Institute for Political Science, Centre for Social Sciences Budapest, Budapest, Hungary; ^6^Faculty of Education and Psychology, Institute of Intercultural Psychology and Education, ELTE, Eötvös Loránd University, Budapest, Hungary; ^7^Budapest Institute, Budapest, Hungary

**Keywords:** ethnic discrimination, rental housing market, anti-Gypsyism, social intervention, focus group, Hungary, Roma people

## Abstract

The aim of our study is to assess the drivers of discriminatory behaviors of real-estate agents and private landlords toward prospective Roma tenants, relying on qualitative data from Hungary. Though there is a broad literature on the forms and frequency of discrimination, we know much less about the question of *why people discriminate*. Previous research suggests that discrimination on the basis of ethnicity is widespread in Hungary. To understand the drivers of discrimination, we analyzed: (a) the sources and justifications of discrimination of Roma people on the rental housing market among real-estate agents and private landlords, the actors making decisions about tenants (b) mapped the social embeddedness of discrimination, and (c) assessed the resilience of discriminatory intentions by analyzing the reactions to a 3-min advocacy video showing discrimination of Roma people on the rental housing market. We conducted and analyzed five online group discussions with 18 real estate agents and landlords advertising properties for rent in different regions of the country. Our qualitative study revealed that discrimination of Roma people is understood to be a widespread and socially acceptable practice driven by the need to avoid risks attributed to Roma tenants based on widely held stereotypes about them. We identified certain specificities in the justification and argumentation strategies of real-estate agents in comparison to private landlords. By providing counter-information presenting the perspective of Roma tenants, negative views could be challenged on the emotional level and also by shifting the group dynamics, strengthening the viewpoint of those without prejudice. We discuss our findings with regards to the possibilities of interventions against discrimination in societies in which neither social norms nor state institutions expect the equal treatment of the members of ethnic minority groups.

## 1. Introduction

A 27-year-old, soft-spoken man calls in response to an advertisement for a rental apartment in a medium-sized city in Southern Hungary. After discussing some details about the apartment, he says: “*I would just like to add that I am of Roma origin, I hope this is not a problem*.” After a short silence, the friendly, female real-estate agent responds: “*I will need to ask the owner if the property is still available*.” When following up later, the caller was turned down yet again.^2^ Discrimination of Roma people in Hungary is a wide-spread practice in various fields of their everyday lives, including education, healthcare, and the labor market (ECRI, 2023). Though the above example is far from an isolated case, in Hungary there has only been scarce research evidence collected on the discrimination of Roma tenants on the rental housing market (Udvari et al., 2008; Balogi and Papadopulosz, 2020).

Discrimination of ethnic minorities is a severe social problem and has been the focus of the social sciences for the past decades. In the present study, we focus on the unequal treatment of Roma people in the Hungarian rental housing market, which has not been researched systematically in this context yet. Our article presents the qualitative analysis of five focus group discussions with the participation of real estate agents and private landlords. Our goal was to explore the underlying mechanisms of their discriminatory decisions in the selection of future tenants.

Long-term accommodation rental, starting with an online advertisement, can be understood as an economically risky, high-stakes offline experience, therefore trust between users is to be seen as a crucial resource—similarly to the case of “peer-to-peer” short-term home-sharing platforms (Tjaden et al., 2018). This is because the initial communication between platform users is online, or through the phone, followed by a high-stakes offline experience. In the case of long-term home rentals, the owner rents out their valuable property, adding to the significance of trust between the two parties. To establish and maintain trust online, many platforms adopt review and reputation systems, along with using personal photos (Ert et al., 2016). By receiving information about prospective tenants, landlords can reduce risks. According to the concept of statistical discrimination, estimations about the trustworthiness of applicants are made based on observable characteristics of applicants, such as ethnic origin or gender. Providing information about individual applicants can reduce this type of discrimination. Home sharing platforms, like CouchSurfing and Airbnb find such information crucial for building trust online (Liu, 2012; Király and Dén-Nagy, 2014).

In Western democracies, civil rights legislation has been built on the principle of prohibiting discrimination based on protected characteristics (such as gender, ethnicity, or disability) since the second World War. Anti-discrimination legislation has a long tradition, and the European Union has followed this by introducing relevant regulations in the early 2000s [most importantly *The Race Equality Directive (2000/43/EC)* 2000^3^; *Employment Directive (2000/78/EC)* 2000^4^ that became legally binding in 2009]. In 2012, the EU Charter of Fundamental Rights was adopted, which includes Article 21, expressly forbidding discrimination, including that based on disability and ethnic origin.^5^ In Hungary, Act CXXV (2003) on Equal Treatment and the Promotion of Equal Opportunities in line with the above EU Council Directives prohibits discrimination based on ethnicity, though studies show that there is a lack of mechanisms preventing the discrimination of Roma people (ECRI, 2023). For the sake of simplicity in the present paper we understand discrimination based on Altman (2011, p. 1) definition, as “acts, practices, or policies that impose a relative disadvantage on persons based on their membership in a salient social group”. To put it more simply, discrimination involves treating someone as less deserving and denying them access to a service that they should be entitled to.

Our paper proceeds in the following way: Section 3 provides a brief review of the main sociological and social psychological explanations of discrimination. Section 4 focuses on the most relevant economic explanations of discrimination in rental markets. Section 5 offers a concise overview of the social context in which our empirical study was carried out. Section 6 explains the materials and methods we used in our research. Section 7 discusses the main results of our qualitative study. Finally, Section 8 is dedicated to the conclusions and recommendations for future research.

## 2. Sociological and social psychological explanations of discrimination

Discrimination is not only prohibited by law but is also morally wrong. Therefore, people often seek justifications for their discriminatory behavior. Social norms and intergroup stereotypes can both serve as such justifications (Crandall et al., 2022). As people are motivated to appear non-prejudiced, they may suppress its expression to conform to the general egalitarian norms (Crandall and Esleman, 2003). However, suppression does not guarantee prejudice and discrimination free treatment or the disappearance of intergroup bias in society. Bias can emerge in subtle (Pettigrew and Meertens, 1995), implicit (Banaji and Greenwald, 1994), or aversive forms (Dovidio and Gaertner, 2000), which all refer to more unconscious, automatic, disguised expressions of prejudice that are more difficult to detect, but maintain social inequalities. Despite the prevalence of anti-prejudice norms in most Western democracies, there are social conditions that facilitate overt prejudice expression against some groups even within these contexts (Kende and McGarty, 2019).

Nevertheless, most studies on social norms and prejudice were carried out in intergroup contexts in which norms clearly prohibit overt expressions of prejudice.

This is not the case in Hungary where prejudice against minority groups, such as the Roma and immigrants are widespread and socially sanctioned. According to recent surveys, there is no social consensus regarding the non-acceptance of ethnic prejudices (Örkény and Váradi, 2010; Kende et al., 2017), thus deeply ingrained anti-Roma prejudice could pose a significant obstacle to integration efforts. At the same time, not everyone agrees with the dominant social norms. While one might disagree with the content of the norm, non-conforming opinions are less likely to be voiced (Hornsey et al., 2003). According to the Theory of the Spiral of Silence, people who believe to have a minority opinion often suppress their views because of a fear of isolation and exclusion which may limit the plurality of opinions and lead to the dominant opinion's misperception as a social norm (Noelle-Neumann, 1974). Following this logic, by giving a platform to opinion-minorities, the hegemony of the majority-view can be questioned. Social-norms interventions aim at achieving social change by breaking down the dominance of certain norms and offering alternative norms (Paluck, 2009; Paluck et al., 2010; Prentice and Paluck, 2020).

Though prejudice and stereotypes are difficult to change, perspective-taking is a promising avenue for delivering such information in an effective way. Perspective taking aims to reduce inter-group prejudice by encouraging majority group members to empathize with a member of the minority group (Todd and Galinsky, 2014). Even brief personal interactions that promote perspective taking with an outgroup member can lead to lasting attitude change (see e.g., Broockman and Kalla, 2016). These findings were further supported by a study conducted in Hungary among adolescents who showed lower levels of anti-Roma prejudice following the participation in an online game designed to depict the perspective of a Romani adolescent (Simonovits et al., 2018).

## 3. Economic explanations of discrimination in the rental markets

Compared to measuring attitudes, the measurement of discrimination poses additional challenges. Since the beginning of the 2000s, correspondence studies assessing the levels of discrimination in the labor, housing, and informal markets, focusing on a large variety of perceived traits, have been mushrooming. One important limitation of the experimental approach is that these studies typically rely on binary outcome variables, e.g. whether a job applicant or a prospective tenant is called back or not and therefore, have limitations in answering the questions of “*why an individual discriminates*” (Banerjee and Duflo, 2017)? The fundamental problem with the use of binary outcomes is that they can only detect coarse discrimination (i.e., refusals and ignoring). Subtle mechanisms of discrimination may be better measured by content analysis of responses or other qualitative methods (see e.g., Farmaki and Kladou, 2020).

The theory of *statistical discrimination*, originally developed by Phelps (1972) and Arrow (1973), posits that in the absence of direct information about a certain fact or ability, a decision-maker would substitute group averages. Discrimination in the labor market may exist because employers do not have sufficient information about an applicant's ability, therefore they base their employment decisions on the applicant's visible features of race, ethnicity, age, and gender.

In contrast, the basic idea of *taste-based discrimination* is that certain people do not like members of certain out-groups so they do not want to be in contact with them, unless they are somehow compensated. Becker (1971) revealed three forms of taste-based discrimination. Originally the three basic forms of discriminatory behaviors were developed within the labor market context, namely: employers' co-workers' (who prefer not to work with people from minority groups because they have a preference against them) and additionally, discriminatory customers (who are only willing to purchase a product from minority workers if they can pay less for it). This typology can easily be transferred to the housing market context, differentiating among agents' neighbors' and customers' (Ondrich et al., 2003; Ahmed et al., 2010; Auspurg et al., 2019) and additionally owners' taste-based discrimination (Verstraete and Verhaeghe, 2020).

Midtbøen (2014) highlights that the key distinction between taste-based discrimination and statistical discrimination lies in the concept of rationality. In the labor market context excluding the most qualified job applicant due to their observable group characteristics (e.g., race or gender) is not economically efficient. However, hiring decisions based on estimated group productivity are considered rational, albeit still discriminatory, responses to the uncertainties and limited information inherent in the labor market's hiring process.

These classic models are also criticized by Bohren et al. (2019) and Barron et al. (2020), arguing that the original taxonomy (statistical vs. taste-based) is too narrow, and a new, more dynamic approach is needed, differentiating between biases based on explicit and implicit beliefs. However, the above typology can be applied to the context of housing discrimination. In their recent meta-analysis, Auspurg et al. (2019) gathered 71 studies conducted in North America and Europe to investigate statistical discrimination in the rental housing market. The authors argued that discrimination is a significant factor in ethnic inequalities in rental housing markets. Furthermore, Flage's meta-analysis—based on correspondence studies from 25 separate studies conducted in OECD countries between 2006 and 2017—indicated that both gender and ethnic discrimination are present in the rental housing market across OECD countries. Applicants with minority-sounding names (particularly with Arabic or Muslim sounding names), as well as men experienced higher levels of discrimination compared to clients with majority sounding names or women.

More than two decades ago, Massey and Lundy (2001) carried out a large-scale field experimental study, in the USA, (in the Philadelphia metropolitan area), employing university students as testers from different social and racial groups. In the late 1990s, answering machines were a standard way of communication with real-estate agents. The authors concluded that racial discrimination against Black tenants was significant and was often aggravated by class and gender. Furthermore, they also highlighted that current technology enables landlords to discriminate based on race without the need for personal contact or experiencing any discomfort or inconvenience. Nevertheless, Quillian et al. (2020), found evidence of a decrease in housing discrimination from the late 1970s to the present—based on 16 field experiments of housing discrimination and 19 observational studies of mortgage lending in the USA. Feagin and Sikes (1994) also underlined that voice-based discrimination exists in the US rental market, by providing anecdotal evidence of discrimination against middle-class black applicants during their apartment search. Based on their qualitative study, the authors concluded that middle-class black apartment-seekers developed painful strategies such as intentionally using a “white-sounding” voice, either their own or a friend's, to prevent racial discrimination.

Even though the company implemented an anti-discrimination policy in 2016 (called Open Doors policy^6^, see McMahon, 2016), various forms of discrimination, such as direct (refusal of booking requests from guests belonging to certain minority groups) or indirect discrimination (creating their property listings that indirectly exclude specific guest groups) continue to persist on the platform (Farmaki and Kladou, 2020). In line with these results, several Budapest-based Airbnb hosts also expressed the view that excluding certain types of guests was not equivalent to discrimination, rather, it was perceived as a necessary tactic for safeguarding the property and alleviating potential risks (Simonovits et al., 2021b). These forms of discriminatory practices can be labeled as *digital discrimination*, which means that even though digital transactions offer the potential to minimize the exchange of undesirable or unnecessary information (in contrast to face-to-face interactions), many of these platforms, in order to enhance interpersonal trust, encourage their users to display personal information about themselves; this facilitation of personal information sharing can then enable discrimination based on observable group characteristics (Edelman and Luca, 2014). This issue is highly relevant for the current research, considering that selecting a trustworthy tenant may be justified as a risk-reducing strategy from the hosts' point of view (for the Airbnb equivalent, in the Hungarian context, see Simonovits et al., 2021b).

To sum it up, experimental studies are crucial in distinguishing between statistical- and taste-based discrimination (for meta-analysis on ethnic discrimination in the labor market see Zschirnt and Ruedin, 2016). At the same time, these studies are unable to shed light on the mechanisms that lead to discriminatory behavior of individuals. Therefore, our study aims to fill this gap by: (i) investigating the drivers and justification mechanisms of discrimination of Roma people by real-estate agents and private landlords, (ii) mapping the social embeddedness of discrimination, and (iii) assessing the rigidity of discriminatory intentions by analyzing the reaction to a 3-min advocacy video showing the discrimination experiences of Roma people on the rental housing market.

## 4. The social context: discrimination of Roma people in Hungary

The Roma minority is the largest transnational ethnic minority in Europe (10–12% of the population, Bernát and Messing, 2016).^7^ Most Roma people live in the countries of East-Central and South-East Europe. Roma minorities in the Central-Eastern European region have been a historically underprivileged minority for centuries living in disadvantageous socio-economic conditions and being often the targets of various forms of discrimination until today. International organizations, including the EU Fundamental Rights Agency (FRA), as well as both governmental and non-governmental bodies, have reported instances of unequal treatment of Roma people (Koszeghy, 2009; Balogi and Papadopulosz, 2020; Király et al., 2021). This unequal treatment occurs at both institutional and interpersonal levels (FRA, 2016). They are facing prejudice (Enyedi et al., 2004; Örkény and Váradi, 2010; Kende et al., 2017; Váradi et al., 2021), as well as mistreatment and hate speech (Pálosi et al., 2007; Sík and Simonovits, 2008; Simonovits et al., 2018) in all European countries, including Hungary (Miller et al., 2008; FRA, 2016, 2018; Kende et al., 2021), where in 2008 and 2009 five Roma adults and one child were murdered in a series of racially motivated killings. Furthermore, in Hungary, members of the Roma minority are often targets of various forms of discrimination, most importantly on the labor market, but also in their access to education and health care (FRA, 2018), during police stop and search practices (Miller et al., 2008), and on the housing market (FRA, 2016). Discrimination of the Roma by local governments in Hungary was recently proven by correspondence studies (Csomor et al., 2021; Simonovits et al., 2021a).

In Hungary, Roma people have significantly worse living conditions and limited access to social and public services compared to non-Roma individuals (Ladányi and Szelényi, 2003; Koszeghy, 2009; FRA, 2016; HCSO, 2021). According to the Hungarian Central Statistical Office (HCSO), 60% of the Roma were at risk of poverty and social exclusion in 2021 (HCSO, 2021). A report by the Fundamental Rights Agency (FRA) found that 22% of Roma in Hungary faced housing discrimination (FRA, 2016). Therefore, it is important to examine their treatment in the housing market by the predominantly non-Roma owners and real-estate agents.

In comparison to other developed countries, there is a lack of systematic scientific evidence about ethnic discrimination in the housing market in Hungary. As housing is a basic human need, housing discrimination is not merely a legal issue, but it also has significant moral, social, political, and economic consequences. In this study, we focus specifically on the discrimination of Roma people in the long-term rental housing market in Hungary. The rental housing market is of interest due to current changing trends and growth of the market itself. While Hungary has traditionally been a country of “super-homeownership”, with 86% of the population living in homes they own, the private rental sector has grown significantly from 2.5% in 2003 to 28% in 2015 (Csizmady and Koszeghy, 2022, p. 3). This growth is particularly significant among lower-income households who may not have other options than to rent (Csizmady and Koszeghy, 2022, p. 3). Furthermore, there is a generational shift in the housing market as young adults are increasingly turning to rental housing. In 1999, only 10.2% of people under the age of 36 were present on the private rental market, compared to 30.3% in 2015, with 37.7% of young people in Budapest living in rentals (Csizmady and Koszeghy, 2022, p. 8). Therefore, given the growth and relevance of the private rental market, it is important to conduct research in this area. In Hungary, homes are advertised either directly by their owners, or by real-estate agents commissioned by the owners. Thus we focus on these two groups in our study.

## 5. Materials and methods

This focus group research from Hungary was part of an ongoing project focusing on discrimination against Roma people in the rental housing market and developing and testing interventions to reduce discrimination. Group discussions were selected as the most suitable data collection approach to study how discrimination is talked about and justified in groups (Roller and Lavrakas, 2015). As opposed to individual interviews, group discussions allow researchers to collect information on social interactions, shedding light on the social embeddedness of the studied phenomena, an important focus of our study. Based on previous studies, showing that in the Hungarian context social norms do not seem to ban prejudiced expressions against Roma in the public, we anticipated that participants would be ready to openly discuss their attitudes and practices in relation to Roma tenants (Kende et al., 2017). Discussions were organized online (on the Zoom platform) in order to include participants from different parts of the country, following the guidelines of online group discussions (Poliandri et al., 2023).^8^ The study was explorative and the conversations were guided by three main questions, including a prompt: (i) attributes of ideal and non-ideal tenants, (ii) discrimination of Roma people, (iii) taking the perspective of Roma people based on a prompt in the form of a short documentary film.^9^ Some questions were initially answered individually using the Mentimeter platform to prevent participants from influencing each other. Written responses were then aggregated and presented to participants in word clouds and were discussed by the group. Otherwise, participants were rarely interrupted. The discussions were led by one moderator (the first author of this paper) with the presence of one research assistant responsible for all technical tasks.

In order to test the resilience of anti-Roma attitudes, at around halfway through the discussions, we presented participants with a prompt in the form of a 3.5 min documentary film depicting five young Roma adults trying and failing to find homes to rent. They recorded their experiences of discrimination and were then interviewed about these on camera. The video presents discrimination through the perspective of Roma people, offering an alternative to the dominant discourse among majority society, showing the emotional burden and existential consequences Roma people are faced with.^10^

### 5.1. Recruitment and sampling strategy

**Figure 1:**
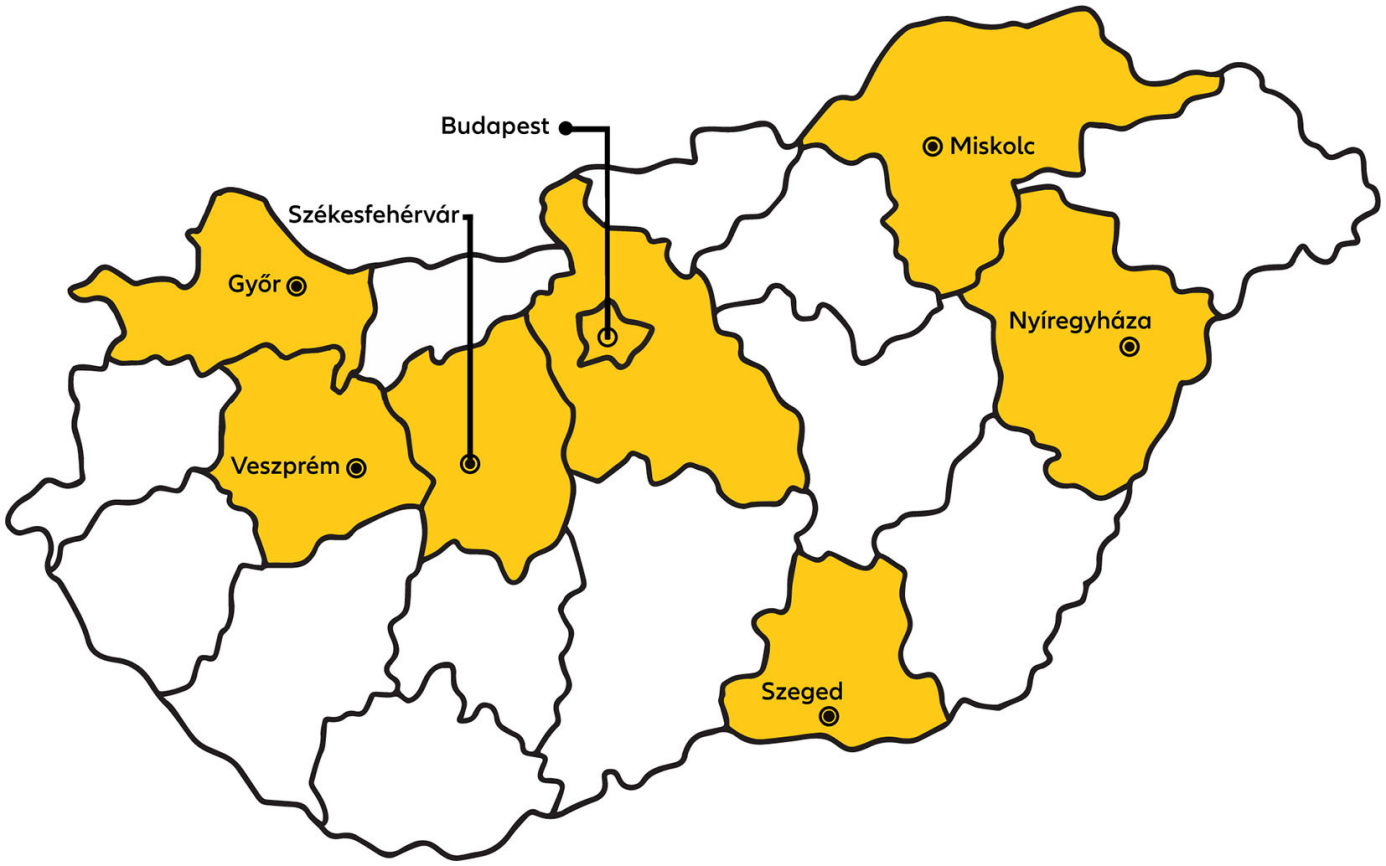
Counties and cities in which participants advertise homes for rent.

Participants of our study were people advertising homes for rent during the period of data collection (1 April−18 May, 2022) on the largest online advertising platform, including real-estate agents (professionals) and private homeowners. We drew a sample randomly selecting participants advertising in the capital city and other large cities, as 85% of rental homes were advertised in these types of municipalities (HCSO, 2023) and this also provided for regional diversity (see Figure 1).

Participants were contacted through the phone and were invited to participate in a study about their experiences on the real-estate market. Interested candidates received an email with information about data protection, anonymity, and all technical details of the study, including a consent form. Participants were offered a voucher worth €9 for their participation.

Altogether five focus groups with 18 participants were conducted (Table 1). In regards to the composition of the groups, two were mixed, and included agents as well as owners, while three were homogeneous, one with only agents, and two with only owners. For each discussion, six to eight participants confirmed their participation and eventually the discussions had two to five participants.^11^ Discussions lasted from 55 to 80 min with most of them lasting for about 70 min.

**Table 1:** Composition of focus groups.

	**Group 1**	**Group 2**	**Group 3**	**Group 4**	**Group 5**
Number of participants	5	3	3	2	5
Number of real estate agents	2	0	2	0	5
Number of private owners	3	3	1	2	0
Number of male participants	3	2	2	1	4
Number of female participants	2	1	1	1	1
Region of property/office	Borsod-Abaúj-Zemplén County, Budapest, Gyor-Moson-Sopron County, Pest County	Budapest, Csongrád-Csanád County	Budapest, Fejér Country, Gyor-Moson-Sopron County	Fejér County, Veszprém County	Hajdú-Bihar County

### 5.2. Data production and analysis

**Figure 2:**
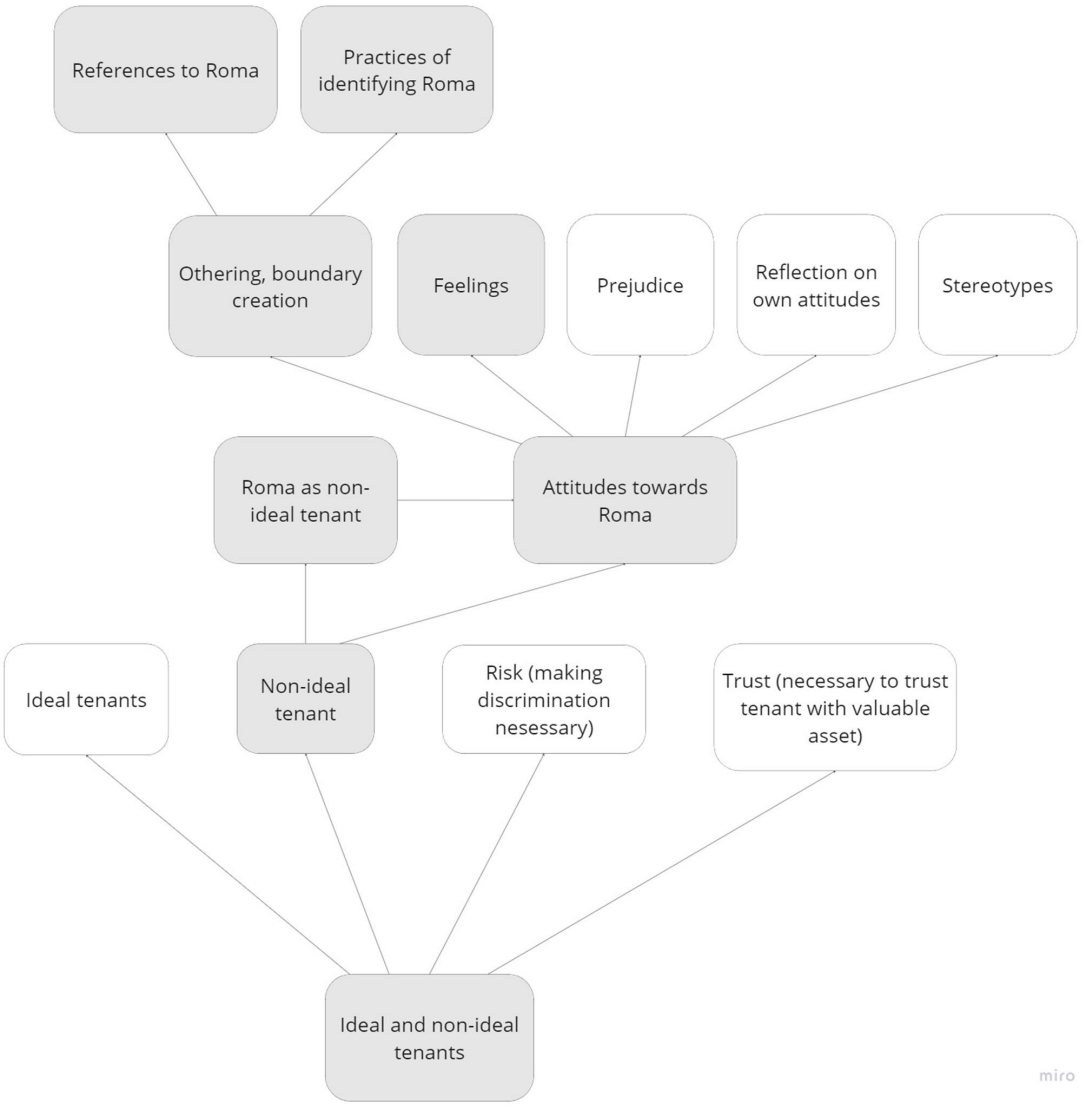
Code map related to attitudes toward Roma.

**Figure 3:**
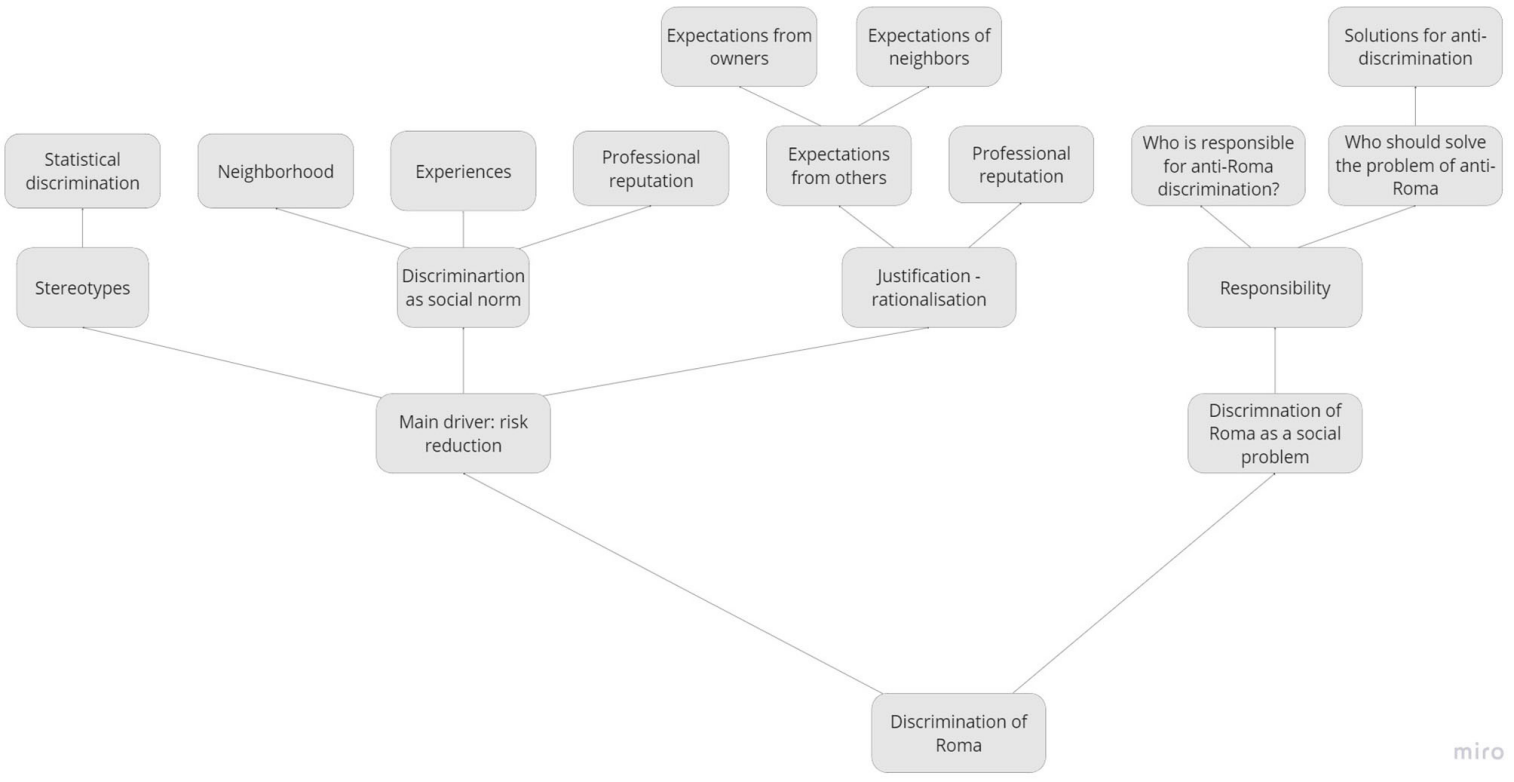
Code map related to discrimination.

**Figure 4:**
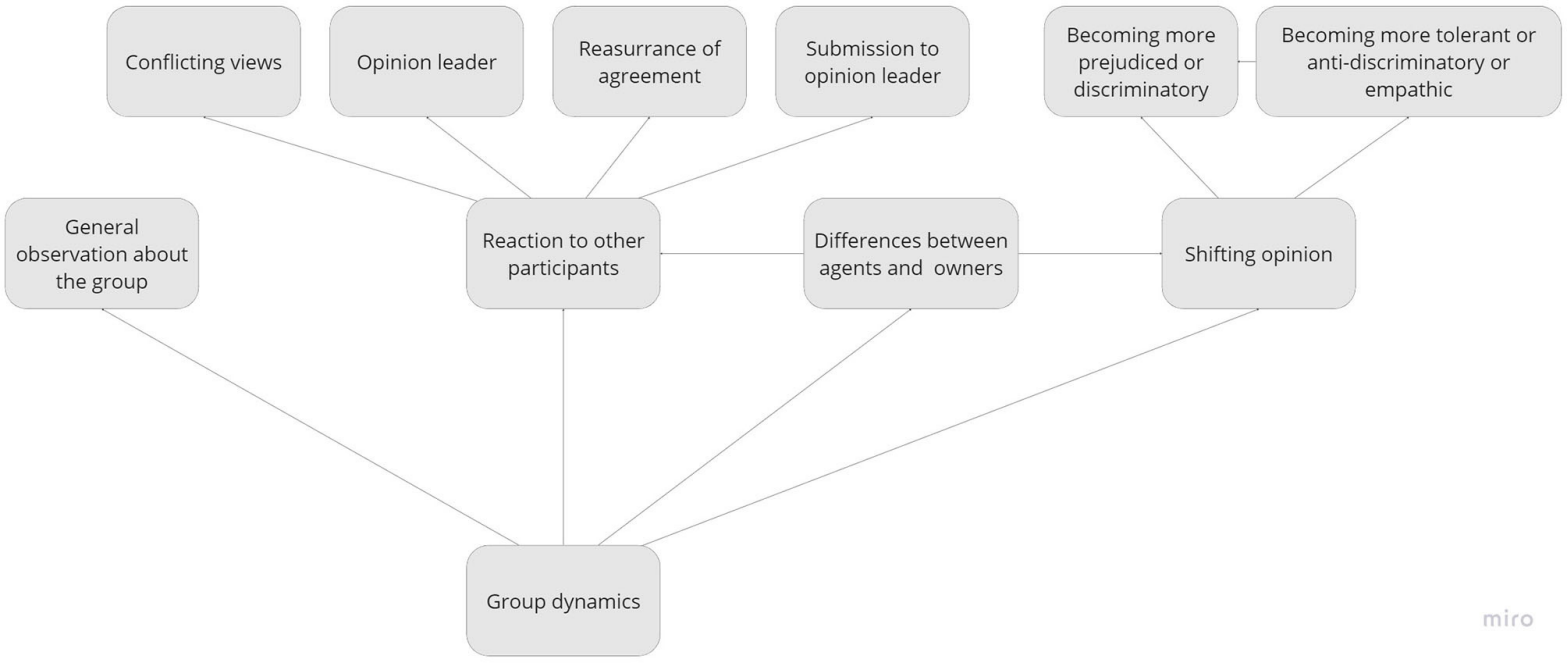
Code map of the group dynamics of the discussions.

**Figure 5:**
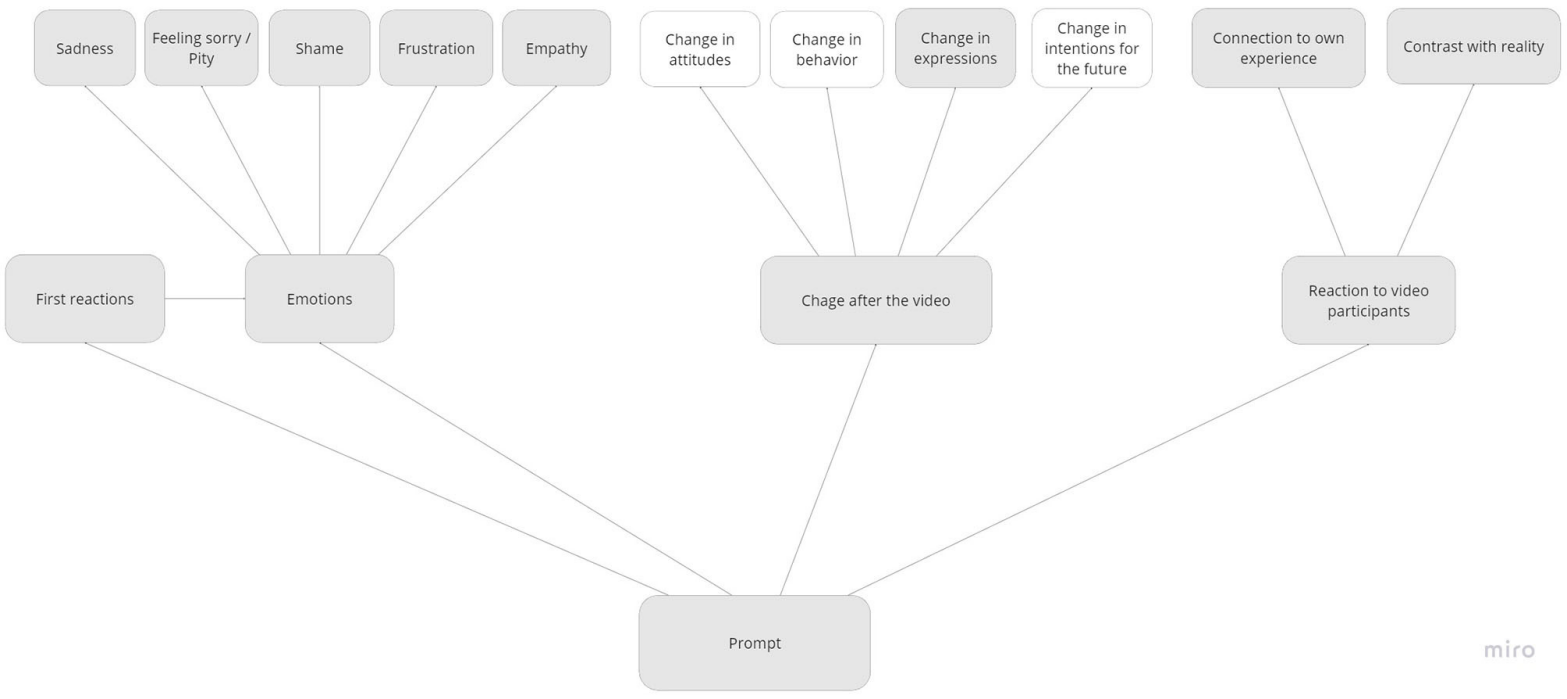
Code map of the effect of the prompt.

All discussions were recorded on Zoom, transcribed verbatim (excluding technical instructions) and analyzed in NVivo in combination with the written responses from Mentimeter. The first and second author of this article and one research assistant carried out a qualitative textual content analysis (Mayring, 2014) taking a phenomenological-interpretative perspective (Moustakas, 1994). A preliminary data analysis was carried out first, taking an inductive/bottom-up approach, developing descriptive codes emerging from the reading of the text, which was then combined with a deductive/top-down approach developing a final codebook of theory-driven, interpretative categories, grouping thematically related codes hierarchically (Poliandri et al., 2023).^12^ The codebook consisted of the following main (second-level) code maps: ideal and non-ideal tenants (Figure 2), discrimination (see Figure 3), group dynamics (see Figure 4) and the effect of the prompt (see Figure 5).

## 6. Results

### 6.1. Discrimination

#### 6.1.1. Categorizing Roma as “Risky Tenants”

To test whether Roma people would be mentioned as problematic tenants, the subject of Roma tenants was not introduced directly by the moderator. As we expected, the topic came up spontaneously after asking participants to list the attributes of ideal and non-ideal tenants and explain their relevance. Roma tenants and how to avoid them became a central topic of four out of five discussions spontaneously and provided a window through which references to Roma, othering mechanisms, and stereotyping Roma tenants could be observed in the context of a semi-public discourse.^13^

We identified a number of ways participants referred to Roma people and signaled their difference from the ingroup. References were made to their appearance, mostly their darker complexion (“*our dark-skinned brothers*”), their origin and ethnic belonging (“*different nationality; minority; racial origin*”), and their typical dialect (“*not talking normally*”). When referring to Roma people, participants sometimes used the neutral term, “Roma”, and, in some cases, the more derogatory term, “Gypsy”. When participants did not explicitly mention these two terms, they used coded language assuming that others will know who they have in mind. These included terms like “category C clients”^14^, or codes referring to their looks and customs, wearing traditional skirts and owning certain types of cars, similar to findings on racial profiling by the police (Miller et al., 2008). These coded references to Roma shed light on the ambiguity and unease people feel when discussing this topic, assuming that others share their negative attitudes, while being aware of the expectation not to appear as racist. In most cases, the representations of Roma people portrayed them as a unanimous mass, rather than as individuals, in line with the findings of Bernáth and Messing about the media-representation of Roma in Hungary (Messing and Bernáth, 2017, p. 458). Classifying a potential client as Roma was predominantly done based on linguistic signifiers. Participants across focus groups mentioned dialect in relation to ideal tenants and explained that the distinguishable dialect or linguistic style of Roma people immediately gives away their ethnic belonging, and potentially disqualifies them from viewing the apartment.

“*Coming back to the Roma issue, if I hear on the phone that they are Roma, I usually ask them to confirm it and then I apologize, and tell them that this is a condition that no Roma person can move into the apartment and then the discussion is over.”* (Pál, male, agent)^15^

“*I also wrote dialect, I mean our dark-skinned brothers. You can tell from the phone how someone speaks, so I mean specifically for their dialect. So it's a big filter, it's quite a big filter (...).”* (Gyula, male, owner)

In line with the qualitative results of Feagin and Sikes (1994) as well as the experimental results of Massey and Lundy (2001) we found qualitative evidence of voice-based discrimination, labeled as “racial screening” (p. 454). It is a significant finding of our qualitative analysis that phone-based communication still serves as an important channel of ethnic discrimination in Hungary.

### 6.2. Drivers and justifications of discrimination

#### 6.2.1. Risk-reduction: the key driver of discrimination

Renting out real-estate is a high-risk, long-term economic transaction for owners, so it is not surprising that reducing risks is a key driver for both private owners and real-estate agents when selecting tenants (cf. Cohen and Sundararajan, 2015; Tjaden et al., 2018; Simonovits et al., 2021b). When asked at the beginning of the discussions to list the attributes of ideal and non-ideal tenants in writing, we obtained a long list of positive and negative characteristics, mirroring the varied dimensions along which real-estate agents and private owners attempt to reduce the risks tenants might pose to the real-estate and its owner. We grouped these characteristics along four dimensions: (1) financial credibility, (2) socio-cultural background, (3) personality, and (4) number and age of tenants, mentioning different types of risks related to each of these (see Table 2). Decisions about viewing the property or renting them were made along these dimensions driven by a motivation to reduce potential risks posed by the applicants. At a first glance, these attributes do not seem to be linked to ethnicity, but having asked participants to explain what these characteristics refer to and why they found them relevant, Roma ethnicity became a central topic of the discussions and a number of stereotypes about Roma as potentially “risky tenants” could be identified. After having identified risk-reduction as the key driver of discrimination of Roma tenants, we explored how this practice is justified by homeowners and real estate agents.

**Table 2:** Risk reduction strategies along four main dimensions (qualitative results, *N* = 18).

**Dimensions of risk-reduction**	**1. Financial credibility**	**2. Socio-cultural background**	**3. Personality**	**4. Number and age of tenants**
Examples of characteristics and behavior expected from tenants	Has a stable job with good income. Earns well. Is not poor.	Well-organized. Good communication skills. No dialect. Educated. Real Hungarian.	Trustworthy. Intelligent. Friendly. Well-mannered. Well-dressed. Punctual. Respectful.	Not more than two people. No children. Middle-aged adults.
Related risk if applicant does not have these characteristics	Will not pay rent. Will not be able to cover the cost of damage caused in property.	Will not take good care of the property. Will disturb the neighbors. Roma ethnicity.	Will not be cooperative. Will not behave properly.	Will not be possible to expel tenants with children. Property will be overcrowded.

#### 6.2.2. Justifying the discrimination of Roma tenants based on stereotypes

In the first two dimensions of risk-reduction strategies, referring to the financial situation and socio-economic background of Roma, participants equated Roma ethnicity with poverty and a lower socio-cultural status, in line with findings from previous studies. In this case, commonly held stereotypes of Roma not having stable jobs, being criminals, and being poor were mentioned. Participants also referred to the Roma as a group with a distinct culture separate and different from that of non-Roma Hungarians, coupled with a certain type of lifestyle that would not be acceptable for tenants (see e.g., Csepeli, 2010; Kende et al., 2017).

“Unfortunately, with this dialect comes a way of life” (Gergo, male, owner)

Being of Roma ethnicity was also equated with untrustworthy character, disrespect for the property owner, and bad manners when communicating with members of the majority society. The looks of Roma people were also discussed in a negative manner, mentioning that they were not well-dressed, wearing a tracksuit or shorts for viewing the apartments, which were also perceived as signals of disrespect for the agent or the owner. This was discussed in parallel to the stereotype that Roma people do not respect the schedule of those of the majority society and call at improper times.

“Obviously, needless to say that we are all equal, but unfortunately the group of people that was mentioned are not reliable. Of course, we cannot generalize, but unfortunately it is true in general.” (Antal, male, agent)

„Yes, there is a definable group of people who like to make phone calls on Sundays and they are a problem, that's my experience. Maybe P's experience is different, mine is this.

Moderator: And can you explain what group you refer to?

S.: Hm... Let's speak in plain Hungarian: the Gypsies.” (Szabolcs, male, agent)

The stereotype about Roma people being disrespectful of the cultural norms of the non-Roma majority was also linked to the expectation that they would not take good care of the property and would damage it causing financial burden and inconveniences to the owner.

Finally, the stereotype that Roma people have large families was mentioned as a risk as well, suggesting that they would be overcrowding the apartment. One real-estate agent also mentioned that even if the tenants seem trustworthy, they would invite family members for celebrations who would not behave properly. The idea that Roma people have large and unruly families and are likened to animals is not far from a more severe, dehumanized view of the group, which in fact, explicitly emerges in the conversations (for the blatant dehumanization of Roma people, see e.g., Kteily et al., 2015).

“It was just my experience that behind these people there is a family. And obviously, if a large flock of crows appears, either on a weekend or in connection with a christening or whatever, it can cause problems. (...)” (Zsófia, female, agent)

The way these characteristics were discussed served two purposes. First, they were used to mark the differences based on which they are identified as Roma during the first encounter, mostly a phone call. Second, they were used to highlight how Roma people pose tangible threats as tenants.

Participants also voiced their frustration about the process of renting out real-estate in Hungary. The process does not include any risk-reduction, i.e., tenants are not required to provide proof of their financial credibility or recommendation from previous landlords. That is the reason why, due to the lack of objective, factual information about the applicants, real-estate agents, and private landlords, when making decisions about who gets to rent a home, rely on the stereotypes we presented earlier. These are basically presented to serve as substitutes for credible information and are used to establish the level of risk, in line with the mechanism of *statistical discrimination* (defined originally by Phelps, 1972; Arrow, 1973 in the labor market context) that has also been identified in the housing market (see a meta analysis by Auspurg et al., 2019).

Based on the solely negative stereotypes mentioned in connection to Roma people, Roma ethnicity itself was presented as a risk in the rental housing market. This suggests that the discrimination of Roma applicants is beyond statistical discrimination encompassing different forms of *taste-based discrimination*. These observations align with the three forms of the Becker (1971) model mentioned earlier. For instance, in certain instances, real estate agents have stated that they are unable to rent out an apartment to a Roma tenant due to the negative attitudes of the home owner. Similarly, in other cases, discrimination may arise from negative stereotypes held by neighbors. Thus, while participants argued for establishing trustworthiness and making decisions based on facts, they continued to rely on stereotypes when it came to Roma, leading to exclusion of all members of the group, and not allowing for exceptions due to the generalized nature of stereotypes.

#### 6.2.3. Further justifications of the discrimination of Roma tenants

Besides stereotyping, discussed above, we identified three further types of justifications of discrimination participants used: (1) social norms and perceived societal expectations; (2) generalization based on actual or alleged negative experiences with Roma people; (3) specifically real-estate agents' concern for their professional reputation if they allow Roma people to rent out homes through their involvement.

Discrimination of people of Roma origin was considered normal and widespread by participants. This is in line with the findings of previous studies on anti-Roma prejudice being perceived as a social norm in Hungary (Kende et al., 2017; Váradi et al., 2021). On the one hand, discrimination of potential Roma tenants was justified by the perceived expectations of the resident community, and on the other hand, by the perceived expectation of owners. They are both connected to the highly hostile normative context and to giving validity to negative stereotypes about the Roma. To some extent this way of thinking about the resident community can be understood in the framework of Becker (1971), which originally focused on the labor market and implied that discrimination reflects the taste of employers, coworkers, or customers. In this case, neighbors can be matched to the co-workers or clients in the labor market scheme.

“It is the community of residents that first comes to mind. I have an apartment in a condo. If I bring a tenant who does not fit well into that community, I will be judged. So this is also a risk for me.” (Gergo, male, owner)

“The residential community must be taken into account: do not bring people who do not fit into the residential community (...).” (Patrik, male, agent)

In these cases, agents view themselves as “gate-keepers” responsible for maintaining the “white” character of the neighborhood or housing project. This is of course more than a question of ethnic boundary-making, as the perception of the difference between Roma and non-Roma people overlaps with that of cultural and class differences, perceiving Roma as lower-class with a distinct and less-advanced cultural level compared to the non-Roma majority, discussed in the previous section on stereotypes (Csepeli, 2010; Kende et al., 2017).

“You have to be very careful who you rent to. Although, we are only the pre-screeners and obviously it is the owner who decides. And obviously you choose a real estate agent because you trust that this filter will work well.” (Antal, male, agent)

This type of justification, referring to others' expectations, may be a manifestation of conformity, i.e., people following the perceived societal and group norms of their communities. At the same time, it may also be a way to deflect the responsibility for discrimination from oneself and project it onto other members of society. This means that, while participants treat discrimination and unequal treatment of Roma to be common and acceptable, they still find it important not to *appear* as “racists”. Thus, social norms are ambiguous regarding this topic and there seems to be a split between acceptable behavior and how this behavior is to be perceived and justified (see Crandall and Esleman, 2003, p. 414). This contradiction was to be seen in the cases when participants used code words to refer to Roma (discussed earlier), and in cases when, before discussing their practices and justifications of discrimination, started their sentences with some variation of: “*I'm not racist, but…*”

“I consider myself a fairly sensitive, socially sensitive person with high empathy, but at the same time, if you look at the statistics (...). Or no, I don't give a number, but the vast majority [of Roma] are like that and the vast majority conform to stereotypes.” (Gyula, male, owner)

Previous negative experiences with Roma tenants were generalized to the entire Roma community which served as justification for discrimination. These accounts of negative behavior by Roma tenants amplify the perceived risks Roma might pose. The experiences were discussed in a way making it clear that the negative behavior was exclusively due to the Roma ethnicity of the tenants, closely linked with negative stereotypes about the group. The accounts of negative experiences rely on a mix of personal experiences the research participants had and stories they heard from others and, in some cases, anecdotes, or simple presentation of stereotypes as actual experiences of acquaintances.

“After all, they damage the apartment, they only cause problems. They leave a utility bill. We had a friend who said that they took away the toilet bowl. And what can they do with the toilet bowl? That's all.” (Zsófia, female, agent)

While most experiences related to Roma tenants that participants shared in the discussions were negative in nature, there were a number of positive accounts. These, however, in contrast with the negative ones, were not generalized in the same way. They were rather treated as exceptions, resisting change in the pre-existing stereotypes (Johnston, 1996) not making any real difference in how participants intended to treat Roma people as prospective tenants. This mechanism will be discussed in detail in relation to the prompt we used.

“You just can't spend in such a fast-moving market, you can't spend a week with this young Roma couple to make sure they can pay the rent. They might as well spend that week making you believe whatever they want. They will pretend that everything is fine.” (Ambrus, male, agent)

The third justifying mechanism was specific to real-estate agents, who explained that giving a chance to Roma tenants would have negative consequences for their professional reputation. This mechanism is related to perceived norms and societal expectations that real estate agents would be in some kind of a gate-keeper position, not allowing Roma to enter the properties of the non-Roma majority. If they go against this expectation, they may risk their reputation and future commissions. That is why they do not always follow their immediate economic interests and rather invest in their reputation for the longer-term.

“And in the long run, I'm looking at this from a business point of view, I'm going to be working with the owner in the long run, not the tenant.” (Ambrus, male, agent)

“And if I now say that we were here with a Roma yesterday [and the owner was not OK with it], but we would return with another Roma today, then it is clear that he [the owner] will say no. And then I'll make a fool of myself because I can't bring him the tenant he wants.” (Mária, female, agent)

“And very importantly, we live off the trust of the owners. They provide the work (...) and they refer us [to other owners].” (Pál, male, agent)

As it was discussed above, according to Midtbøen (2014) the main difference between taste-based and statistical discrimination is the notion of rationality. Similarly to the labor market context, excluding the “best” (i.e., the most trustworthy and fitting) applicant due to their observable group characteristics (i.e., their Roma ethnicity) is not economically efficient.

### 6.3. Discrimination of Roma as a social problem

In order to understand discrimination of Roma people in a broader sense, not only as an individual act, we analyzed how participants viewed and discussed discrimination as a social problem. There was a broad consensus among participants that discrimination of Roma tenants was a wide-spread, commonly known, and socially acceptable practice in the Hungarian rental housing market. While many of our participants gave detailed accounts of their own discriminatory practices, there was still a common understanding that discrimination is morally wrong, and people should not experience it (see Bohren et al., 2019).

#### 6.3.1. Who is responsible for the discrimination of Roma on the rental housing market?

We identified three groups that were blamed for the discrimination of Roma people: (1) the Roma themselves, (2) Hungarian society in general, and, (3) property owners (only among real-estate agents). In line with previous studies, there was a common understanding that Roma people themselves were to be blamed for their discrimination and unequal treatment when looking for homes to rent (Bernát, 2010). The core of this argumentation was based on the negative stereotypes about Roma people we have discussed earlier. Participants argued that for Roma people these stereotypes serve as the norm they conform to and this validates the discrimination toward all Roma.

“Stereotypes are not formed by accident, so that's basically why I think that this group of people is entirely responsible for the way Hungarians treat them.” (Gyula, male, owner)

Parallel to this, some participants stated that not all Roma were “bad” but a small or large proportion of Roma people were criminals or acted in unacceptable ways, disqualifying all Roma from being considered as tenants. This argument suggested that Roma people need to take collective responsibility for how members of *their* group behaved and therefore, also needed to suffer from the consequences collectively.

“Well, I feel sorry that a minority suffers because of the majority. So, within the Roma, there is an educated group who are now the victims of discrimination, which is practically being directed against them because of a criminal group.” (Szabolcs, male, agent)

By putting the blame on Roma people themselves, participants argued that they should accept being measured by higher standards than non-Roma Hungarians. Thus, Roma applicants were not only expected to accept their unequal treatment, they were also expected to make extra efforts if they wanted to get forward in life. This was a clear reversal of cause and effect, acknowledging the inequality and unfair treatment of Roma people, but blaming them for it and expecting them to overcome this burden without societal support.

“If necessary, they [Roma people] need to be open to admitting that they are starting from a disadvantage. So whichever way you look at it, that's the way it is. They start with a disadvantage in comparison to a Hungarian. They should know that they have to prove themselves.” (Ambrus, male, agent)

Participants also pointed at Hungarian society at large as being responsible for the discrimination of Roma people. Participants claimed that there was a high level of prejudice toward people of Roma origin among the majority society and acknowledged that racism against Roma was deeply-rooted in Hungarian society, leading to discrimination.

“I think that Hungarians are racist. The Hungarian people are terribly racist.” (Antal, male, agent)

“Hungarian society is really very prejudiced. We don't even give a chance to those who would do better or do everything flawlessly if we gave them the opportunity.” (Alexa, female, owner)

Nevertheless, participants distanced themselves from the general population as actual agents of discrimination. They did so by referring to an abstract group of people, without specifying the actors who discriminate within society, creating the image of society itself being a distant and coherent actor, unrelated to participants.

Real-estate agents tended to rationalize their own discriminatory behavior by the requirements of property owners and claimed that owners did not accept Roma tenants and, therefore, they were to blame for the discrimination of Roma.

“There is only one thing that matters here. The owner decides.” (Ambrus, male, agent)

“There are owners who refuse Gypsy tenants. This I have to accept.” (Imre, male, agent)

“But really, we represent the owners, and if the owner has the prejudice that he doesn't want a Roma, we won't take a Roma.” (Mária, female, agent)

In summary, on the one hand, participants explicitly stated that discrimination was morally wrong, on the other hand, real-estate agents and landlords alike disclosed how they themselves discriminated against potential tenants of Roma origin. To shield themselves from the weight of immoral—let alone illegal—actions, participants applied the above-described mechanisms to shift the responsibility of discrimination onto others, such as Roma people, society at large, or owners of the properties. At the same time, when it came to the evaluation of their own practices of discrimination, we identified another strategy participants used to free themselves from the weight of moral wrongdoing. We identified the mechanism of victim blaming both among agents and private landlords claiming that rather than perpetrators, they are the victims of the Roma. Victim blaming tends to occur more frequently toward members of out-groups than in-groups and to be used as a justification for prejudice (e.g., De Keersmaecker and Roets, 2020). Participants explained that since Roma people were to be blamed for discrimination, they put the ones advertising the rental properties into a difficult position needing to go through the uncomfortable act of turning down Roma applicants, i.e., carrying out discrimination. Here, seeing discrimination as normal and justified clashes with the notion that discrimination was morally wrong, therefore not comfortable *for the members of majority society*. As if Roma were putting an extra burden on advertisers by responding to their ads and showing interest in renting the advertised properties.

“Although it's not polite to say ‘yes, because you're a Gypsy', it's the truth and it's unpleasant for us. And they shouldn't be offended by it, because they know that this is the case and it's not unexpected, it's not the first time they're hearing this in their lives, that they're being rejected because they're a Gypsy. They've probably been through this several times since childhood. Of course I understand that it's bad for them, it would be bad for me too. I think it's a complete dead end. We are suffering side by side here. It's bad for them, it's bad for us... it's inefficient, it's economically damaging, in all kinds of ways.” (Gyula, male, owner)

We also identified another approach to justifying participants' own practices of discrimination, in which they took some degree of responsibility for their actions, but explained that, for practical reasons, they could not avoid discriminating against Roma, since this would require an unrealistic amount of extra effort, due to the risks Roma people generally pose.

Finally, discussing feelings in relation to Roma applicants being discriminated against, it became clear that even if empathy was present, it would be too costly to feel sorry for individual applicants, and therefore, it was wiser not to have such feelings.

“Obviously, it's not pleasant, and we can psychologize it, but anyway, in real estate, especially if you're dealing with rentals, if you put yourself in the shoes of every single person, you can burn out quickly.” (Szabolcs, male, agent)

#### 6.3.2. Proposed solutions to discrimination

While research participants openly discussed and justified discriminatory practices, they also morally condemned the existence of discrimination in the Hungarian rental housing market, and voiced their frustration for the lack of mechanisms that would prevent it. Based on their suggestions for improvement, we identified two strands of recommendations. The first suggestion focused on Roma people, expecting them to change their behavior, the second is aiming at altering the way the rental housing market works.

The requirement for the change of the behavior of Roma is again based on the notion that Roma are responsible for their own discrimination. When participants argued about individual change, it was very much in line with the logic of statistical discrimination, expecting Roma to prove that they were different from the stereotypical image of “risky Roma tenants”. This proof could be in the form of different documents or a very high level of transparency during the rental process. The stereotype about Roma not being trustworthy and not telling the truth were the ones needed to be disproved by individual applicants. It was also made clear that Roma people were expected to disclose their ethnicity as part of the process and not to try to pass as non-Roma, even if they did not look or talk like stereotypical Roma do.

“Anyway, for me it would be very positive if somebody came up to me and said, ‘Listen, I'm a Roma.' So it's already giving confidence that he's really taking a stand, standing up, speaking nicely, telling the truth, not trying to deceive, being honest. So that gives a lot of positives. And I'd be most reassured if he introduced himself. And told us a little bit about himself. Anyway, if he wants to convince me, he has to tell me about his upbringing, his family, his schooling. What motivates him, why he works, what his life's purpose is. So, if I could see in him the things that even among Hungarians there are very few people like that, I would immediately say: okay listen, that was totally convincing, we'll write a strict contract. If there are no problems, then of course you can stay as long as you want to.” (Gyula, male, owner)

Some participants described their desire for the rental housing market to be more regulated. They gave us examples from abroad, where tenants were required to provide information about their financial credibility, references from previous landlords and about their work. Such strict regulations of the housing market are clearly in line with the reduction of statistical discrimination. In this case, basically participants require objective information from all potential tenants in order to eliminate the need to rely on stereotypes.

“Because in the West, for example, you can only rent an apartment like that [by submitting financial documents]. Because in my case, if I ask for an employer's certificate, I would get laughed at, or if the applicant really means it, he might [submit these]. But I have no serious possibility to check the tenant to whom I give possession of an apartment. Whereas in the West it is how they do it. I have cousins living in Berlin who are also renting out an apartment. There, if the tenant doesn't pay for a month, the employer deducts it from his salary and transfers it. So it is much stricter, much better regulated on both sides and very transparent. It's not like here, where they don't pay for months, they damage the apartment and you practically can't evict them. I really, really wish we had this in Hungary.” (Alexa, female, owner)

“I would also add that this is not done in Hungary, but abroad it is standard practice to ask for the contact details of the landlord of the previous apartment and a letter from the employer to check if it is true what they say. And it's a crucial thing what the previous owner says, what kind of tenant he was, what condition he left the apartment in, whether he paid on time. But that's not the norm here.” (Júlia, female, owner)

In relation to solving the problem of discrimination, participants did not mention any sort of legal measures or regulations banning this practice, although discrimination is clearly banned by legislation in Hungary. In one case a participant asked the researcher leading the discussion, whether the discriminatory practices he described were in fact legal. Other than that, participants did not seem to be aware of the legal aspects of discrimination and did not demonstrate any fear of being caught for openly carrying out such practices.

Neither landlords, nor real estate agents mentioned the responsibility of the state, municipalities, or other public bodies or institutions in preventing discrimination. Since discrimination is normalized in the rental market, even if it was seen as somewhat immoral and uncomfortable for all concerned parties, participants did not express that they would be in favor of effective regulations banning this practice.

Finally, even though they discussed in detail that discrimination was a severe problem, and understood its immoral nature and the related injustices, participants did not feel that they themselves could be the solution to this problem. They did not think that they were in a position of power allowing or not allowing Roma people to find a home. The argument in relation to this was clearly related to the normalcy of discrimination, asking the question: why should I be the one solving this problem, if everyone is discriminating against Roma people and neither the state, nor other public institutions do anything to stop this? To sum up, we found that participants did not feel responsible for the problem of discrimination, nor have they attempted to take responsibility for contributing to its solution.

### 6.4. Group dynamics

#### 6.4.1. Understanding discrimination as a social construct

Group discussions offer an ample opportunity to understand how certain phenomena are constructed in a semi-public setting, shedding light on the social nature of these constructs (Roller and Lavrakas, 2015, p. 105). In the discussions, it was clearly observable that the expression of negativity toward Roma could be openly discussed in a semi-public setting without fearing repercussions from others. Furthermore, practices of discrimination were also shared in the group without fear of judgement, or legal consequences.

By the use of code-words to refer to Roma at the beginning of the discussions, participants had first detected the manner in which negativity toward Roma can be voiced, but were confident that other participants would be able to decode their language and not question their position toward Roma. As the discussions unfolded, participants constructed a shared understanding of how Roma were to be seen as actors on the rental market in a similar manner across four out of five discussion groups. As participants got to hear each other's views on this subject, there was a detectable degree of mutual sympathy among people sharing similarly negative attitudes, experiences, and practices making them feel close to each other, as suggested by expressing agreement through little remarks, such as “*I agree, I had similar experiences…*”. Thus, the expressions of anti-Roma attitudes could be understood to function as a common ground along which people could connect (Székelyi et al., 2001).

While there was a certain degree of variance in the level of prejudice expressed by participants within the groups, even those who declared not to harbor prejudice and who did not intend to discriminate, did not dare to openly question the dominance of the position of those who used racist language and advocated for discrimination, until they had been presented with the prompt. In the mixed groups in which both real-estate agents and private landlords were present, the dynamics unfolded in the manner that agents presented themselves as professionals, sharing their professional views with the “lay owners”. Real-estate agents in these discussion groups expressed more prejudice and all recounted their discriminatory practices. Among private owners, there was more variation on their attitudes toward Roma with some participants not expressing prejudice nor intending to discriminate. It was also observable that in some of the groups one or two real-estate agents became the opinion-leaders, usually responding first to the moderator's questions. They intended to convince other participants about the necessity of discrimination, and were not confronted with different views, others more or less accepted their opinions. Those who expressed such views have related to the ones without prejudice with contempt and placed them on a lower level on the hierarchies of professionalism and life experience.

In one focus group, two male real-estate agents, in reaction to a female private owner's statement that she would not discriminate against Roma tenants, used their negative experiences to convince this participant that it would be in her own interest not to allow Roma people to rent her apartment. This example is a clear display of the power of the accounts of negative experiences as a justification for discrimination. All participants except this female owner, who was not willing to discriminate against Roma tenants, formed a consensus that even those who have not yet had negative experiences will have them eventually. This example clearly illustrates how discussion participants anticipated that the only possible direction for the change in attitudes and behavior toward Roma tenants was negative.

There have been some observable differences between the group dynamics related to the size and composition of the groups. In smaller groups, participants were less likely to talk spontaneously and needed more direct questions. However, larger groups had more lively and free-flowing conversations. In Groups 1 and 5, participants initiated conversations by sharing their own experiences and opinions and asked others about their experiences.

In Groups 2 and 4, only owners, while in Group 5 only agents were present. In these homogenous groups, there were less conflicting views. This was especially true when discussing justifications of discrimination and the responsibility of reducing discrimination. Still, we identified some conflicting views in Group 5, in which only real estate agents from the same city participated. This might have happened both due to professional competition and due to the larger number of participants. However, heterogeneous groups had somewhat different dynamics. Real estate agents, especially with more experience, tended to share their views more openly than private owners. Moreover, agents shared their conflicting views in cases when they disagreed with owners with less experience. In these cases, agents verified their experiences with their knowledge collected throughout the years.

### 6.5. Taking the perspective of Roma—reactions to the prompt

To measure the degree of robustness and resilience of the attitudes toward Roma and the reactions to information counter to the dominant anti-Roma attitudes, we presented a prompt to participants in the form of a 3.5-min documentary video showing the experiences of five young Roma persons trying to find homes for rent and being turned-down. During the discussions, the moderator did not bring up the topic of Roma as potential tenants before showing the video, but this topic came up spontaneously in four out of five discussions before the video, in relation to the attributes of ideal and non-ideal tenants. Right after watching the video, participants were asked to write down their first thoughts and the feelings they had while watching it, and then these were discussed among the group.

Regarding the feelings evoked by the video, participants immediately mentioned sadness, pity, shame, and frustration in writing. In their written evaluations of the video, participants also mentioned that it was “*effective, true, and disturbing*”. When asked to start talking about the video and discuss what they had written, interestingly, mostly those participants spoke first who had been less dominant in the previous parts of the discussion and who had not expressed negativity toward Roma. Thus, the video offered support to those who were otherwise in the opinion-minority by questioning the perceived dominance of the anti-Roma norm, as if the Spiral of Silence (Noelle-Neumann, 1974) had been broken by showing an alternative to the norm of prejudice. The first quote below comes from one of the private owners who earlier expressed no intention to discriminate against Roma. The second quote comes from a real-estate agent, who openly expressed her discriminatory practices before watching the video and found it difficult to speak after the video.

“I think the film is quite impressive, so it definitely touches me.” (Gergo, male, owner)

“I find it difficult to speak, because if this is an isolated case, then I feel very sorry for those who obviously find themselves facing prejudice. It is true that there is obviously a reason, because 9 out of 10 cases act according to the stereotype. Unfortunately, it is very difficult to speak now. (…).” (Erzsébet, female, agent)

This change in group dynamics lasted until the end of the discussions and it was also observable that participants who previously expressed prejudice and discriminatory practices overtly started to use a more politically correct language toward Roma. While the hostile content had not changed, the tone of language did, with references to Roma becoming less hostile, e.g., by starting to use the non-derogatory term, Roma, or refusing to directly refer to them.

When it came to discussing feelings, we found a mix of reactions among participants. It was clear that they had been touched by the video and empathized with the five young Roma people in the film. While some participants expressed their feelings of sadness and pity in the discussion and showed empathy toward the persons in the video, for many, it seemed to be difficult to accept and express such feelings and they had the need to counter their own feelings evoked by the video with various strategies of discrediting the video itself.

The dominant pattern was that participants first expressed that they felt sorry about the plight of the individuals in the film, not questioning the authenticity of their experiences, as these exactly mirrored the discriminatory practices described by the participants before watching the video. This was followed by some kind of counter-statement discrediting the video and its effect by those who had expressed overt prejudice beforehand. One of these discrediting strategies was to question the prototypicality of the Roma in the video, stating that they were not in line with the stereotypes, thus the video did not present a realistic picture of Roma in general. This strategy was coupled with some mentions of probabilities about how uncommon it was for Roma to be like the ones in the video, with “*one in ten*” or “*one in a 100*” who would be “*this decent*”. This mechanism was similar to when intergroup contact did not reduce prejudice as the individual positive encounter was not generalized, often due to the lack of supporting norms (Lantos et al., 2018).

“I would have put three Roma in there like the ones whom we here have all rejected because they damaged the apartment, left utilities unpaid, took away the toilet bowl.... I feel very sorry for these people, but even without a video I feel very sorry for those who are excluded, but this is just a small slice of the cake.” (Zsófia, female, agent)

The second discrediting mechanism was related to participants' feeling of being held responsible for the discrimination of Roma by the video. They defended their actions by giving counter-evidence about how Roma tenants *really were*. In relation to the feeling of being held responsible, some participants also made general statements about the severity of this problem in Hungary and about the normalcy of unequal treatment of Roma, as discussed earlier.

“[I feel] emotional. It's a polarizing video that can evoke sympathy, but also resistance to why they [Roma people] should be pitied. I wrote earlier that it's their responsibility, but I was generalizing. I did not mean the ones in the film, so to speak, when I gave that answer. (...) I feel sympathy for them [the ones in the film], I feel sorry for them.” (Gyula, male, owner)

The third strategy made it clear that discrimination is an economic necessity, and one may feel sorry for the individual being discriminated against, but cannot make decisions based on emotions.

“So what I can say is that emotionally it's definitely an unfortunate situation. Personally I feel sorry, professionally I don't have a choice.” (Ambrus, male, agent)

The fourth mechanism was a simple disregard of what was presented in the video and instead of discussing it or the feelings it evoked, participants listed accounts of bad experiences with Roma. When asked to discuss their reactions to the video, participants refused to do that and started to reiterate and, in some cases, even amplify their negative experiences with Roma tenants.

The prompt presenting an alternative view of Roma, countering stereotypes and making the injustices tangible and personalized, affected participants which led to emotional and verbal reactions ranging on a broad spectrum. Sadness, pity, and empathy were the dominant feelings, and the prompt reversed the dynamics of expressions of prejudice being previously dominant in the groups. At the same time, participants with robust anti-Roma attitudes seemed resilient to counterfactual arguments but were still touched on the emotional level. It is an open question whether and how the effect of such counter-information could be translated into change in attitudes and behavior.

## 7. Conclusion and recommendations

Our findings clearly demonstrate that the discrimination of Roma people on the rental housing market in Hungary is understood to be a widespread and socially acceptable practice. Discrimination is driven by stereotypes presenting Roma as “risky tenants” and justified by social norms, relying on the expectations of local residents, neighbors and the owners of the homes. Lack of information was identified as a driver for discrimination, though besides the mechanisms of statistical discrimination, taste-based discrimination, i.e., the rejection of all Roma tenants due to their ethnicity, was also observable. Roma people were identified as being responsible for their own discrimination and were, at the same time, expected to solve this problem. Consequently, perpetrators of discrimination did not see themselves as potential actors in bringing about change or finding a solution.

Our findings suggest that real-estate agents consider discrimination against Roma people as a “professional necessity” that is closely linked to their professional reputations and long-term economic interests. Attitudes and practices of non-professional private landlords were more fluid and varied, though they did not question the professional judgment of agents concerning Roma tenants.

While prejudice and discriminatory behavior were blatant and rigid, when presented with the perspective of Roma tenants' experiences, participants were touched on an emotional level, expressing sadness, pity, and empathy. Furthermore, it changed the group dynamics, as non-prejudiced participants became more confident in expressing their views, while those with strong prejudice changed their tone and expressed their views about Roma with less hostile language.

It should be taken into consideration that our sample had a limited size and female participants were underrepresented. In our view, a larger qualitative follow-up study with multiple focus groups at various locations within Hungary, applying a similar design could give robustness to our findings. Participation in the discussions was evaluated as a positive experience by all, except for one person writing in a follow-up email that he felt disappointed about not knowing in advance that Roma-related issues were to be discussed. Other participants said that they found the discussions interesting and were glad to talk about issues that are rarely discussed. Effects of the discussion, and especially those of the short documentary might last beyond the discussion.

Relying on thematic analysis, we identified certain inconsistencies and contradictions that could serve as starting points for intervention against the discrimination of Roma people. First, we found that even those who had expressed blatant prejudice and openly discussed their discriminatory practices, found it important not to *appear* racist. Second, when social norms and expectations of certain reference groups were used as justifications for discrimination, participants have been convinced of anti-Roma prejudice and behavior to be the unquestioned norm. Once presented with the prompt, participants not endorsing this norm were ready to express their contrary view, because of the emergence of an alternative norm. Finally, there was a consensus among participants that discrimination is “a bad thing”.

Based on these points, we propose the following recommendations: we find it important to focus on norms-based interventions, specifically, strengthening the norm of non-prejudice, the norm of the wrongfulness of discrimination and the declaration of ethnicity-based discrimination being an act of *racism*. At the same time, the prompt we had worked with also brought promising results and could serve as the basis for intervention in two different ways. First, it may be implemented directly within the rental process. Given that the effect might be short-term, it may be most effective if implemented in the situation in which decisions about tenants are made, i.e., during the process of advertising homes on online platforms. For this, the collaboration between academics and market actors seems to be the way forward. Second, given the promising effect on group dynamics, the film may serve as an effective starting point for a discussion on discrimination, bringing the perspective of Roma people to the forefront and questioning the dominance of the norm of prejudice. Finally, it is important to match specific interventions with specific actors, designing different types of interventions for real-estate agents, questioning the professionality of discrimination, and for private landlords, enhancing the acceptance of Roma people as tenants.

At the same time, it is important to acknowledge the responsibility of state actors and public institutions in preventing and sanctioning discrimination against ethnic minorities. Regrettably, in Hungary in 2023, the state appears to be absent in addressing the issue of discrimination. Consequently, our policy recommendations are expected to have limited impact unless societal norms that tolerate the mistreatment of Roma people are dismantled in cooperation with public authorities.

We are still hopeful that the implementation of our suggestions can become vehicles for social change and that in the future, the soft-spoken Roma man from Southern Hungary along with all other Roma people will not find it necessary to ask if their ethnic origin poses a problem when looking for a home to rent.

## Data availability statement

The raw data supporting the conclusions of this article will be made available by the authors upon request, without undue reservation.

## Ethics statement

The studies involving human participants were reviewed and approved by ELTE Faculty of Education and Psychology, under Ethical Approval Number 2020/196. The patients/participants provided their written informed consent to participate in this study. Written informed consent was obtained from the individual(s) for the publication of any potentially identifiable images or data included in this article.

## Author contributions

LV designed the research. LV and BSz collected and analyzed the data. LV, BSi, and AK participated in the conceptualization of the paper. LV, BSi, BSz, AK, GS, and JB participated in the writing of the paper. JB directed the film used as the prompt and wrote the note on the creation of the film. All authors contributed to the article and approved the submitted version.

## Footnotes

^2^This scene is from the documentary film used as a prompt in our study.

^3^The Race Equality Directive, no. Council Directive (2000/43/EC), The Council of the European Union (2000). Available online at: https://eur-lex.europa.eu/legal-content/EN/TXT/HTML/?uri=CELEX:32000L0043.

^4^Employment Directive (2000/78/EC), no. Council Directive (2000/78/EC), The Council of the European Union. (2020). Available online at: https://eur-lex.europa.eu/legal-content/EN/TXT/PDF/?uri=CELEX:32000L0078.

^5^Charter of Fundamental Rights of the European Union (2012/C 326/02), no. 2012/C 326/02, European Parliament (2012). Available online at: https://eur-lex.europa.eu/legal-content/EN/TXT/PDF/?uri=CELEX:C2012/326/02.

^6^Open Doors policy has been effective since October 2016, requiring that users of the platform (both hosts and guests) treat all fellow members with respect and without judgment, regardless of their group characteristics such as age, religion, national origin, etc. (McMahon, 2016).

^7^In the present paper, we have chosen to use the terms “Roma” and “people of Romani origin” interchangeably based on the decision made by the Roma community at the First World Romani Congress in 1971 (Kenrick, 1971) to self-define as Roma. The term originates from Romanes and means “people”. We consider it essential to clarify our use of terminology as researchers of non-Romani origin, as we must be conscious of our position in society and use language that is not offensive or loaded with negative connotations toward people of Romani origin.

^8^Organizing the discussions online made it possible to include people from different regions without the need to travel. Furthermore, they could participate from their homes or workplaces, giving them more flexibility and comfort, serving the principle of inclusivity. Given that we drew a sample of people advertising homes for rent online, it was our assumption that participants would be able to navigate online tools and platforms with their smartphones or computers.

^9^The discussion guide is included in Supplementary material 1.

^10^The video is available here: https://www.youtube.com/watch?v=WgI98QCtqJo. It has been created in the framework of the Misrecognition of Minorities in Europe (MisMiE) project funded by the VW Foundation - an independent research foundation between 2019 and 2021, directed by Jeremy Braverman and with the participation of Ádám Hushegyi, Boglárka Nyúl, Anna Kende, József Pántya, and Luca Váradi. A description of the filmmaking process by the film's director is included in Supplementary material 2.

^11^Withdrawals usually happened at the last moment and were explained by the participants' busy schedules. In case of agents, they often got called away for apartment viewings. In the case of owners, the reason was usually related to unforeseen work-related and childcare duties, especially among female participants.

^12^The codebook is included in Supplementary material 3.

^13^In Discussion Group 4., with two participants, both of whom have been private owners, the topic of Roma people did not come up spontaneously before watching the video together. *Altogether, participants in this group had the lowest level of prejudice against Roma based on the facts that, in contrast to all other groups, participants here have not mentioned Roma people as non-ideal tenants and when explicitly asked about the discrimination of Roma, they have clearly rejected this practice*.

^14^C as reference to the first letter of cigány (Gypsy), while placing the whole group into a qualitatively lower category under category A and B.

^15^Participants' first names have been changed. Quotes were translated by the first author and language edited by the fourth author.

## References

[B1] Act CXXV (2003). On Equal Treatment and the Promotion of Equal Opportunities. Available online at: https://net.jogtar.hu/jogszabaly?docid=a0300125.tv, https://www2.ohchr.org/english/bodies/cescr/docs/e.c.12.hun.3-annex3.pdf (accessed May 15, 2023).

[B2] Ahmed A. Andersson L. Hammarstedt M. (2010). Can discrimination in the housing market be reduced by increasing the information about the applicants? Land Econ. 86, 79–90. 10.3368/le.86.1.79

[B3] Altman A. (2011). “*Discrimination” in The Stanford Encylopedia of Philosophy (Winter 2020 Edition)*. Metaphysics Research Lab, Stanford University. Available online at: https://plato.stanford.edu/entries/discrimination/ (accessed May 15, 2023).

[B4] Arrow K. (1973). “The Theory of Discrimination” in Discrimination in Labor Markets, Princeton (Ashenfelter Orley, Rees Albert: Princeton University Press).

[B5] Auspurg K. Schneck A. Hinz T. (2019). Closed doors everywhere? A meta-analysis of field experiments on ethnic discrimination in rental housing markets. J. Ethnic Migr. Stud. 45, 95–114. 10.1080/1369183X.2018.1489223

[B6] Balogi A. Papadopulosz D. (2020). Romákat éro diszkrimináció a budapesti magánbérleti piacon. Utcáról Lakásba Egyesület. 1–16.

[B7] Banaji M. R. Greenwald A. G. (1994). “Implicit stereotyping and prejudice,” in The psychology of prejudice: The Ontario symposium, eds. M. P. Zanna and J. M. Olson (Hillsdale, NJ: Lawrence Erlbaum Associates) 55–76.

[B8] Banerjee A. V. Duflo E. (2017). Handbook of Economic Field Experiments (Volume 2). North Holland: Elsevier.

[B9] Barron K. Diltmann R. Gehrig S. Schweighofer-Kodritsch S. (2020). Explicit and implicit belief-based gender discrimination: A hiring experiment. WZB Berlin Social Science Center, No. SP II 2020-306. Available online at: https://www.econstor.eu/bitstream/10419/223247/1/1727613708.pdf (accessed May 15, 2023).

[B10] Becker G. S. (1971). The Economics of Discrimination. Chicago: The University of Chicago Press. 10.7208/chicago/9780226041049.001.0001

[B11] Bernát A. (2010). Integráció a fejekben: A romák társadalmi integrációjának érzékelése és megí*télése a lakosság körében*. Kolosi Tamás and Tóth István György: Társadalmi Riport 312–326.

[B12] Bernát A. Messing V. (2016). “Methodological and data infrastructure report on Roma population in the EU,” in GRID [Working Paper MS20.3.] Available online at: https://inclusivegrowth.be/project-output/project-output#Project%20working%20papers (Accessed May 14, 2023).

[B13] Bohren J. A. Imas A. Rosenberg M. (2019). The dynamics of discrimination: theory and evidence. Am. Econ. Rev. 109, 3395–3436. 10.1257/aer.20171829

[B14] Broockman D. Kalla J. (2016). Durably reducing transphobia: A field experiment on door-to-door canvassing. Science 352, 220–224. 10.1126/science.aad9713 27124458

[B15] Cohen M. Sundararajan A. (2015). Self-regulation and innovation in the peer-to-peer sharing economy. Univ. Chicago Law Rev. 82, 116–133.

[B16] Crandall C. S. Esleman A. (2003). A justification-suppression model of the expression and experience of prejudice. Psychol. Bull. 129, 414–446. 10.1037/0033-2909.129.3.414 12784937

[B17] Crandall C. S. Esleman A. O'Biren L. (2022). Social norms and the expression and suppression of prejudice: The struggle for internalization. J. Person. Soc. Psychol. 82, 359–378. 10.1037/0022-3514.82.3.359 11902622

[B18] Csepeli G. (2010). Gypsies and gadje: The perception of Roma in Hungarian society. Centr. Eur. Polit. Sci. Rev. 11, 62–78.

[B19] Csizmady A. Koszeghy L. (2022). ‘Generation Rent' in a super homeownership environment: the case of budapest, Hungary. Sustainability 14, 1–18. 10.3390/su14148929

[B20] Csomor G. Simonovits B. Németh R. (2021). Hivatali diszkrimináció?: Egy online terepkísérlet eredményei (discrimination at local governments? Results of an online field experiment). Szociol. Szemle 31, 4–28. 10.51624/SzocSzemle.2021.1.1

[B21] De Keersmaecker J. Roets A. (2020). All victims are equally innocent, but some are more innocent than others: The role of group membership on victim blaming. Current Psychol. 39, 254–262. 10.1007/s12144-017-9763-9

[B22] Dovidio J. F. Gaertner S. L. (2000). Aversive racism and selection decisions: 1989 and 1999. Psychol. Sci. 11, 315–319. 10.1111/1467-9280.00262 11273391

[B23] ECRI (2023). ECRI Report on Hungary (sixth monitoring cycle). European Commission against Racism and Intolerance, Council of Europe. 1–50. Available online at: https://rm.coe.int/ecri-6th-report-on-hungary-translation-in-hungarian-/1680aa687b (accessed May 15, 2023).

[B24] Edelman B. G. Luca M. (2014). Digital Discrimination: The Case of Airbnb.com. Harvard Business School NOM Unit Working Paper. 10.2139/ssrn.2377353

[B25] Enyedi Z. Fábián Z. Sík E. (2004). “Nottek-e az eloítéletek Magyarországon? Antiszemitizmus, cigányellenesség és xenofóbia változása az elmúlt évtizedben,” in Társadalmi riport, 2004, ed. K. Tamás, T. I. György, V. György (Budapest, Hungary: TÁRKI) 375–399.

[B26] Ert E. Fleischer A. Magen N. (2016). Trust and reputation in the sharing economy: The role of personal photos in Airbnb. Tourism Manag. 55, 62–73. 10.1016/j.tourman.2016.01.013

[B27] Farmaki A. Kladou S. (2020). Why do Airbnb hosts discriminate? Examining the sources and manifestations of discrimination in host practice. J. Hospit. Tourism Manag. 42, 181–189. 10.1016/j.jhtm.2020.01.005

[B28] Feagin J. R. Sikes M. P. (1994). Living with Racism: The Black Middle-Class Experience. Boston: Beacon Press.

[B29] FRA (2016). EU-MIDIS II. Second European Union Minorities and Discrimination Survey. Roma—Selected findings. European Union Agency for Fundamental Rights. 1–52.

[B30] FRA (2018). FRAopinions anti-Gypsyism: Discrimination, harassment and hate crime. European Union Agency for Fundamental Rights. Available online at: https://fra.europa.eu/en/content/fra-opinions-anti-gypsyism (accessed May 15, 2023).

[B31] HCSO (2021). A háztartások életszí*nvonala. A Roma népesség szegénységmutatói*. Hungarian Central Statistical Office. Available online at: https://ksh.hu/s/helyzetkep-2021/#/kiadvany/a-haztartasok-eletszinvonala/a-roma-nepesseg-szegenysegmutatoi (accessed May 15, 2023).

[B32] HCSO (2023). KSH–ingatlan.com-lakbérindex, 2023. Január: Hungarian Central Statistical Office. Available online at: https://www.ksh.hu/s/kiadvanyok/kshingatlancom-lakberindex-2023-januar/index.html (accessed May 15, 2023).

[B33] Hornsey M. Majkut L. Terry D. J. McKimmie B. M. (2003). On being loud and proud: Non-conformity and counter-conformity to group norms. Br. J. Soc. Psychol. 42, 319–335. 10.1348/014466603322438189 14567840

[B34] Johnston L. (1996). Resisting change: Information-seeking and stereotype change. Eur. J. Soc. Psychol. 26, 799–825. 10.1002/(SICI)1099-0992(199609)26:5<799::AID-EJSP796>3.0.CO;2-O

[B35] Kende A. Hadarics M. Bigazzi S. Boza M. Kunst J. R. Lantos N. A.. (2021). The last acceptable prejudice in Europe? Anti-Gypsyism as the obstacle to Roma inclusion. Group Proc. Intergroup Relat. 24, 388–410. 10.1177/1368430220907701

[B36] Kende A. Hadarics M. Lasticov,á B. (2017). Anti-Roma attitudes as expression of dominant social norms in Eastern Europe. Int. J. Interc. Relat. 60, 12–27. 10.1016/j.ijintrel.2017.06.002

[B37] Kende A. McGarty C. (2019). A model for predicting prejudice and stigma expression by understanding target perceptions: the effects of visibility, politicization, responsibility, and entitativity. Eur. J. Soc. Psychol. 49, 839–856. 10.1002/ejsp.2550

[B38] Kenrick D. (1971). The world Romani congress—April 1971. J. Gypsy Lore Soc. 3, 101–108.

[B39] Király G. Dén-Nagy I. (2014). How to explain couchsurfing's success? Szociol. Szemle 24, 32–53.

[B40] Király K. J. Bernáth G. Setét J. (2021). Romák Magyarországon: A diszkirimináció kihí*vásai*. Minority Rights Group Europe (MRGE). 1–28. Available online at: https://minorityrights.org/wp-content/uploads/2021/03/MRG_Rep_RomaHung_HU_Mar21_E.pdf (accessed May 15, 2023).

[B41] Koszeghy L. (2009). Hungary – Housing Conditions of Roma and Travellers [Thematic Study]. RAXEN National Focal Point.

[B42] Kteily N. Bruneau E. G. Waytz A. Cotterill S. (2015). ascent of man: Theoretical and empirical evidence for blatant dehumanization. J. Person. Soc. Psychol. 109, 901–931. 10.1037/pspp0000048 26121523

[B43] Ladányi J. Szelényi I. (2003). Historical Variations in Inter-Ethnic Relations: Toward a Social History of Roma in Csenyéte, 1857–2000. Forschungskolleg: Univ., Kulturwiss. 10.3828/rs.2003.1

[B44] Lantos N. A. Macher J. Kende A. (2018). Eloítélet-csökkentés és mobilizáció a romák érdekében. A Tollfosztás-workshop hatásvizsgálata. Alkalmazott Pszichol. 18, 35–55.

[B45] Liu C. S. (2012). A couchsurfing ethnography: traveling and connection in a commodified world. Inquiries J. Stud. Pulse 4, 2–3.

[B46] Massey D. S. Lundy G. (2001). Use of black english and racial discrimination in urban housing markets. new methods and findings. Urban Affairs Rev. 36, 452–469. 10.1177/10780870122184957

[B47] Mayring P. (2014). Qualitative content analysis: Theoretical foundation, basic procedures and software solution. Social Science Open Access Repository. 10.1007/978-94-017-9181-6_13

[B48] McMahon S. (2016). Airbnb Launches ‘Open Doors' Policy to Combat Discrimination. Smarter Travel Media. Available online at: https://www.smartertravel.com/airbnb-launches-policy-to-combat-discrimination/ (accessed May 15, 2023).

[B49] Messing V. Bernáth G. (2017). Disempowered by the media: Causes and consequences of the lack of media voice of Roma communities. Identities. 24, 650–667. 10.1080/1070289X.2017.1380264

[B50] Midtbøen A. H. (2014). The invisible second generation? Statistical discrimination and immigrant stereotypes in employment processes in norway. J. Ethnic Migr. Stud. 40, 1657–1675. 10.1080/1369183X.2013.847784

[B51] Miller J. Gounev P. Pap A. L. Wagman D. Balogi A. Bezlov T.. (2008). Racism and police stops: adapting US and british debates to continental Europe. Eur. J. Criminol. 5, 161–191. 10.1177/1477370807087641

[B52] Moustakas C. (1994). Phenomenological Research Methods. London: Sage Publications, Inc. 10.4135/9781412995658

[B53] Noelle-Neumann E. (1974). The spiral of silence a theory of public opinion. J. Commun. 24, 43–51. 10.1111/j.1460-2466.1974.tb00367.x

[B54] Ondrich J. Ross S. Yinger J. (2003). Now you see it, now you don't: Why do real estate agents withhold available houses from black customers? Rev. Econ. Stat. 85, 854–873 10.1162/003465303772815772

[B55] Örkény A. Váradi L. (2010). Az eloítéletes gondolkodás társadalmi beágyazottsága, nemzetközi összehasonlításban. Alkalmazott Pszichol. 12, 29–46.

[B56] Pálosi É. Sík E. Simonovits B. (2007). Discrimination in shopping centers. Szociol. Szemle 3, 135–148.

[B57] Paluck E. L. (2009). What's in a norm? Sources and processes of norm change. J. Person. Soc. Psychol. 96, 594–600. 10.1037/a0014688 19254106

[B58] Paluck E. L. Ball L. Poynton C. Siedloff S. (2010). Social Norms Marketing Aimed at Gender Based Violence: A Literature Review and Critical Assessment. New York: International Rescue Committee.

[B59] Pettigrew T. F. Meertens R. W. (1995). Subtle and blatant prejudice in Western Europe. Eur. J. Soc. Psychol. 25, 57–75. 10.1002/ejsp.2420250106

[B60] Phelps E. (1972). The statistical theory of racism and sexism. Am. Econ. Rev. 62, 659–991.

[B61] Poliandri D. Perazzolo M. Pillera G. C. Giampietro L. (2023). Dematerialized participation challenges: Methods and practices for online focus groups. Front. Sociol. 8, 1145264. 10.3389/fsoc.2023.1145264 37091722 PMC10118020

[B62] Prentice D. Paluck E. L. (2020). Engineering social change using social norms: Lessons from the study of collective action. Curr. Opin. Psychol. 35, 138–142. 10.1016/j.copsyc.2020.06.012 32746001

[B63] Quillian L. Lee J. J. Honor,é B. (2020). Racial Discrimination in the U.S. Housing and Mortgage Lending Markets: A Quantitative Review of Trends, 1976–2016. Race Soc. Probl 12, 13–28. 10.1007/s12552-019-09276-x

[B64] Roller M. R. Lavrakas P. J. (2015). Applied Qualitative Research Design: A Total Quality Framework Approach. New York: The Guilford Press.

[B65] Sík E. Simonovits B. (2008). “Egyenlo bánásmód és diszkrimináció” in Társadalmi riport, ed. T. Kolosi, I. G., Tamás (Budapest, Hungary: TÁRKI) 375–399.

[B66] Simonovits B. Zách B. Kondorosy C. (2021b). Participation, trust, and risks associated with peer-to-peer accommodation platforms: How did the COVID-19 crisis affect Airbnb Budapest in 2020? Intersections. East Eur. J. Soc. Polit. 7, 178–200. 10.17356/ieejsp.v7i3.790

[B67] Simonovits G. Kézdi G. Kardos P. (2018). Seeing the world through the other's eye: an online intervention reducing ethnic prejudice. Am. Polit. Sci. Rev. 112, 186–193. 10.1017/S0003055417000478

[B68] Simonovits G. Simonovits B. Víg Á. Hobot P. Németh R. Csomor G. (2021a). Back to normal: The short-lived impact of an online NGO campaign of government discrimination in Hungary. Polit. Sci. Res. Methods 10, 848–856. 10.1017/psrm.2021.55

[B69] Székelyi M. Örkény A. Csepeli G. (2001). Romakép a mai magyar társadalomban. Szociológiai Szemle. 3, 19–46.

[B70] Tjaden J. D. Schwemmer C. Khadjavi M. (2018). Ride with Me—Ethnic Discrimination, Social Markets, and the Sharing Economy. Eur. Sociol. Rev. 34, 418–432. 10.1093/esr/jcy024

[B71] Todd A. R. Galinsky A. D. (2014). Perspective-taking as a strategy for improving intergroup relations: Evidence, mechanisms, and qualifications. Soc. Person. Psychol. Compass 8, 374–387. 10.1111/spc3.12116

[B72] Udvari M. Herczku T. Iványi K. (2008). Civil szervezetek és az antidiszkriminációs törvény végrehajtása. Összefoglaló tanulmány./Non-governmental organisations and the implementation of the anti-discrimination law. A synthesis study./Office for National and Ethnic Minority Rights, Otherness Foundation. Available online at: https://adoc.pub/civil-szervezetek-es-az-antidiszkriminacios-trveny-vegrehajt.html

[B73] Váradi L. Barna I. Németh R. (2021). Whose norms, whose prejudice? The dynamics of perceived group norms and prejudice in new secondary school classes. Front. Psychol. 11, 524547. 10.3389/fpsyg.2020.524547 33488435 PMC7817897

[B74] Verstraete J. Verhaeghe P. P. (2020). Ethnic discrimination upon request? Real estate agents' strategies for discriminatory questions of clients. J. Hous. Built. Environ. 35, 703–721. 10.1007/s10901-019-09721-8

[B75] Zschirnt E. Ruedin D. (2016). Ethnic discrimination in hiring decisions: A meta-analysis of correspondence tests 1990–2015. J. Ethnic Migr. Stud. 42, 1115–1134. 10.1080/1369183X.2015.1133279

