# Supplementary material for: “Personally, I feel sorry, but professionally, I don't have a choice.” Understanding the drivers of anti-Roma discrimination on the rental housing market
Source: Front Sociol. 2023 Jul 18;8:1223205. doi: 10.3389/fsoc.2023.1223205 (PMC10393131; doi:10.3389/fsoc.2023.1223205)
Supplement: Supplementary file 1 [file Data_Sheet_1.docx]

Supplementary Material

“Personally, I feel sorry, but professionally, I don’t have a choice.”^[[1]](#footnote-1)^

Understanding the drivers of anti-Roma discrimination on the rental housing market

Luca Váradi^*^, Blanka Szilasi, Anna Kende, Jeremy Braverman, Gábor Simonovits, Bori Simonovits

*** Correspondence:** Luca Váradi, VaradiL@ceu.edu

## Supplementary Material 1. Group discussion guide

Key To Home Project

Focus group discussions

Conducted in April-May, 2022.

**Discussion guide**

INTRODUCTION

1. *Information about project and use of data:*

- Data is processed anonymously.
- There are no "right and wrong" answers, everyone's opinion is important to us.
- Let’s listen to each other and give everyone a chance to express their views.

1. *Briefly about the research*

- Purpose: part of larger study about the rental housing market. Our aim now is to get the views and experiences of homeowners and real-estate agents about the rental housing market. Later we will watch a video together that is related to this topic and we will discuss it.
- Presentation of the research team (CEU-ELTE PPK).
- Brief description of the focus group method.

1. *Focus group*

- Introduction of focus group moderator and assistant: can we please use first names?
- Online group: we plan a 45-minute discussion.
- Introduction to ZOOM interface (login, mute tools)
- Mentimeter, interactive tool: testing, favorite animal (questions M0, M1)
- Link to the mentimeter will be sent in the ZOOM chat.
- Agreement with Zoom recording.

1. *Mentimeter^[[2]](#footnote-2)^ testing and introduction of participants:*

M0. What is your favorite animal? (test question)

M1. Please introduce yourself. Write anything about yourself that is relevant, especially in relation to the housing market.

- Looking at the word cloud together. Discussion of written responses: is there anything you would like to add?

IDEAL AND NON-IDEAL TENANT

M3. What are the attributes of an ideal tenant? What are you looking for during the first encounter? What would make you decide to invite them for a viewing of the property?

M4. What are the attributes of a non-ideal tenant? What are you looking for during the first encounter? What would make you decide not to invite them for a viewing of the property?

- Looking at the word cloud together. Discussion of written responses: is there anything you would like to add?

PROMPT

*Let's watch the 3-minute video below together.*

M6. What comes to your mind first about the video?

M7. How did you feel during the video? Please list all the emotions you had.

- Looking at the word cloud together. Discussion of written responses: is there anything you would like to add?
- Discussion of the video in general.
- Discussion of feelings.

*Characters in the video shown in Mentimeter:*

M8: What do you think about these individuals?


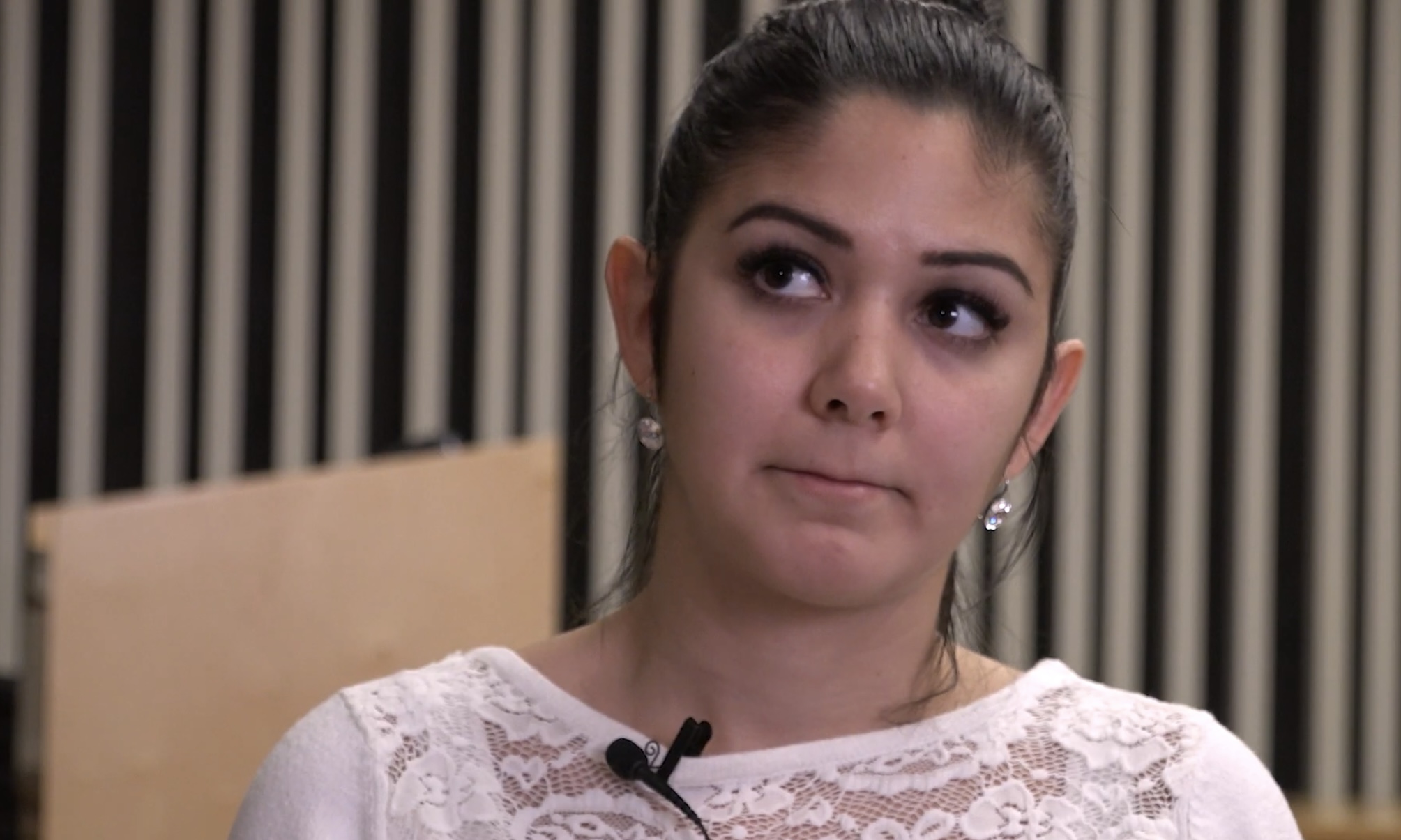

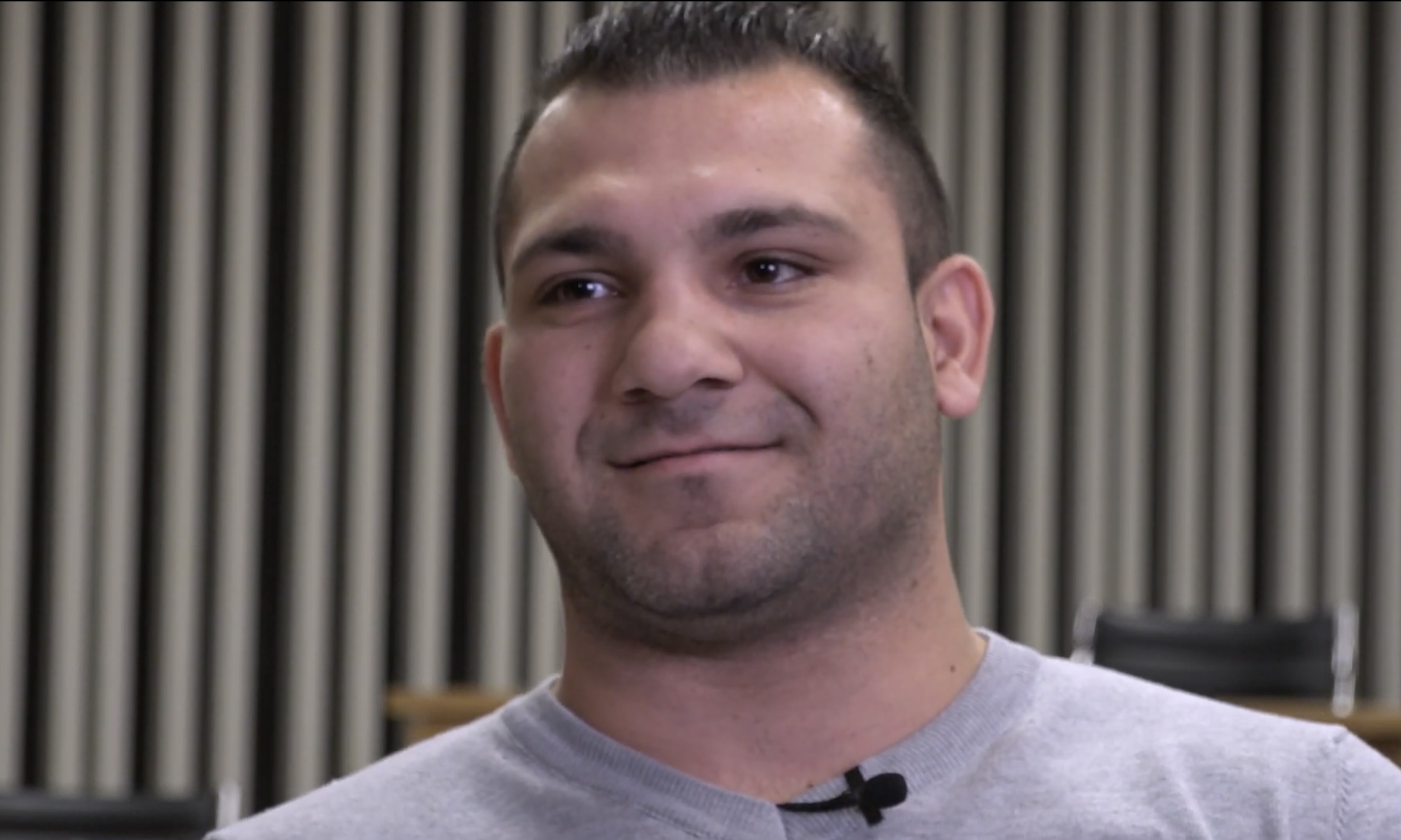

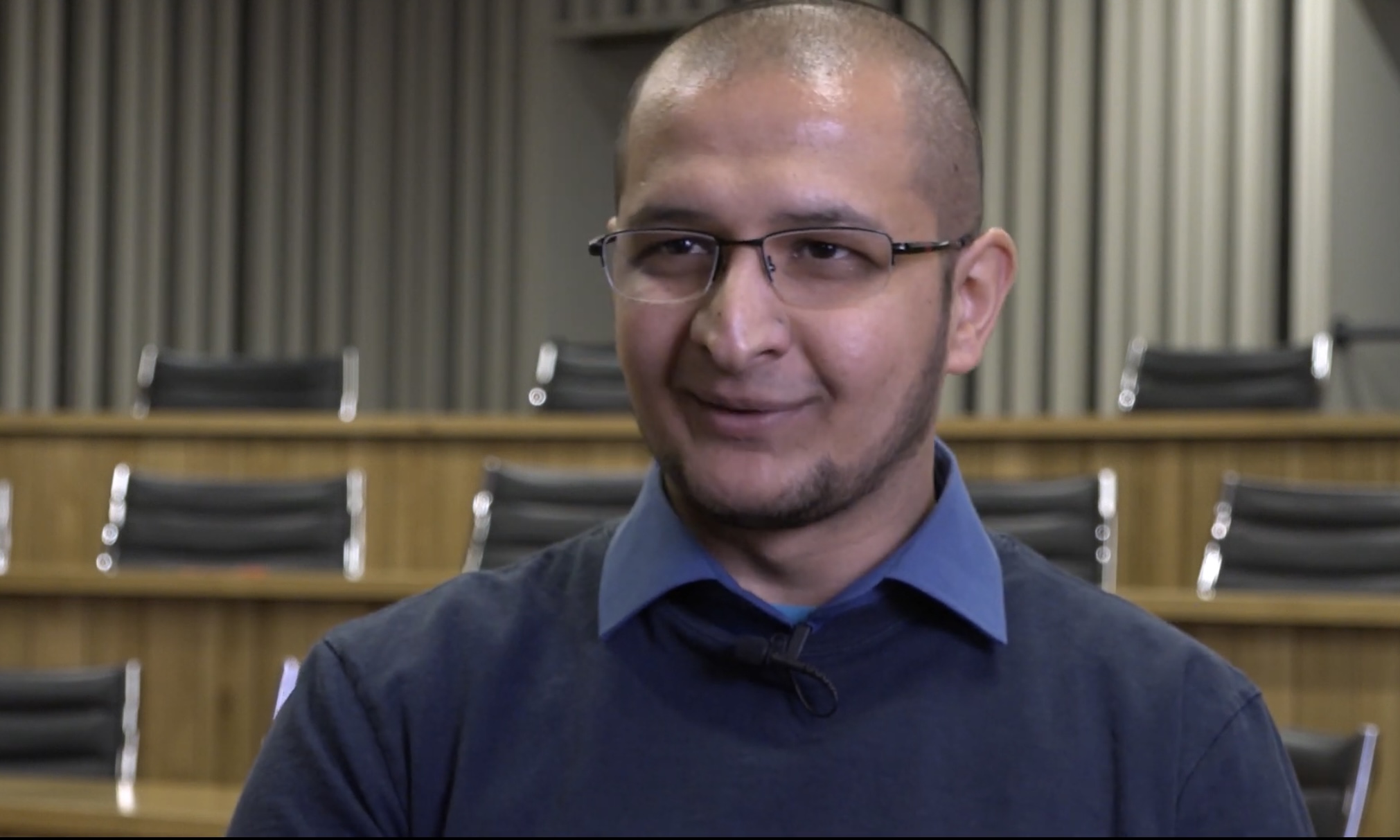

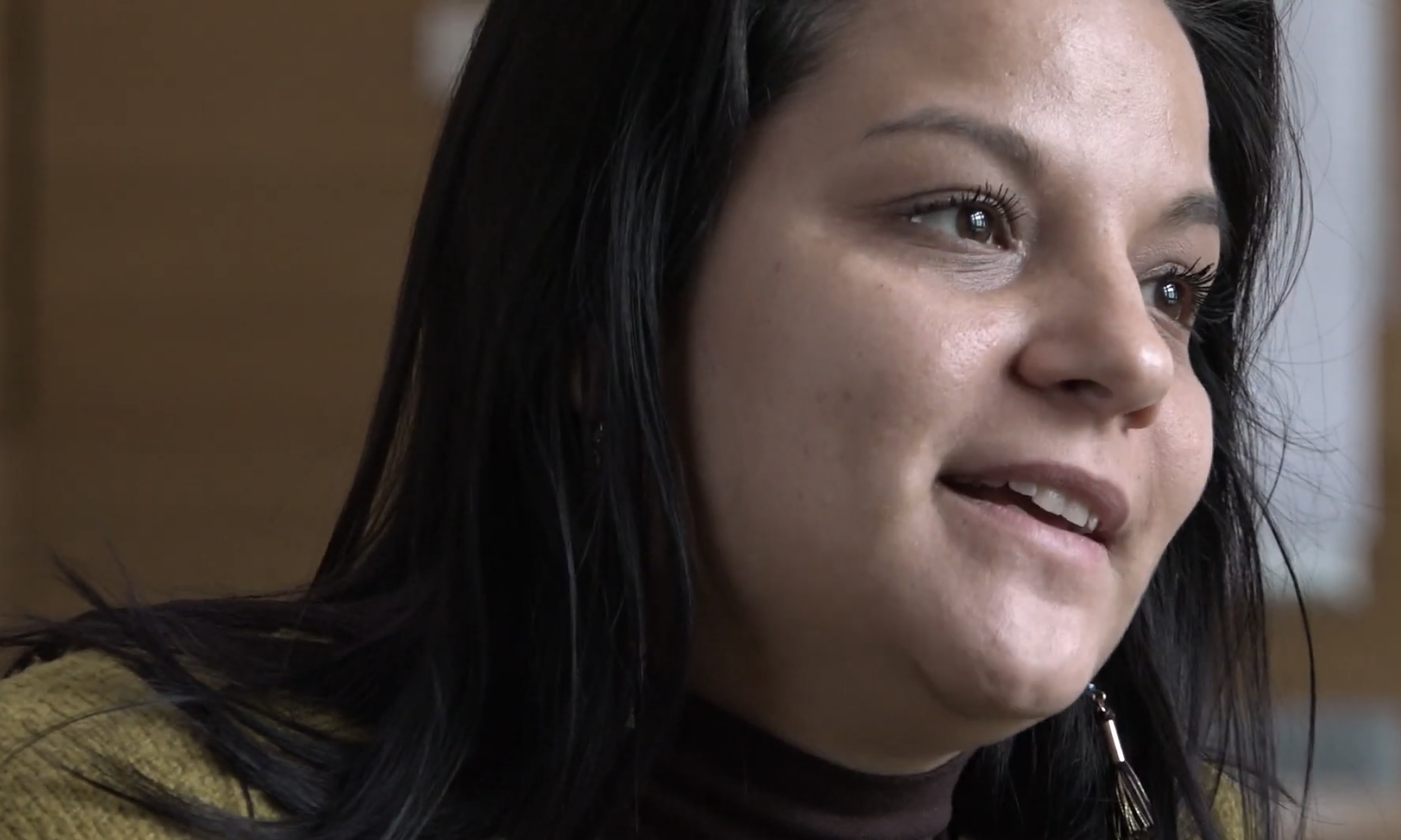

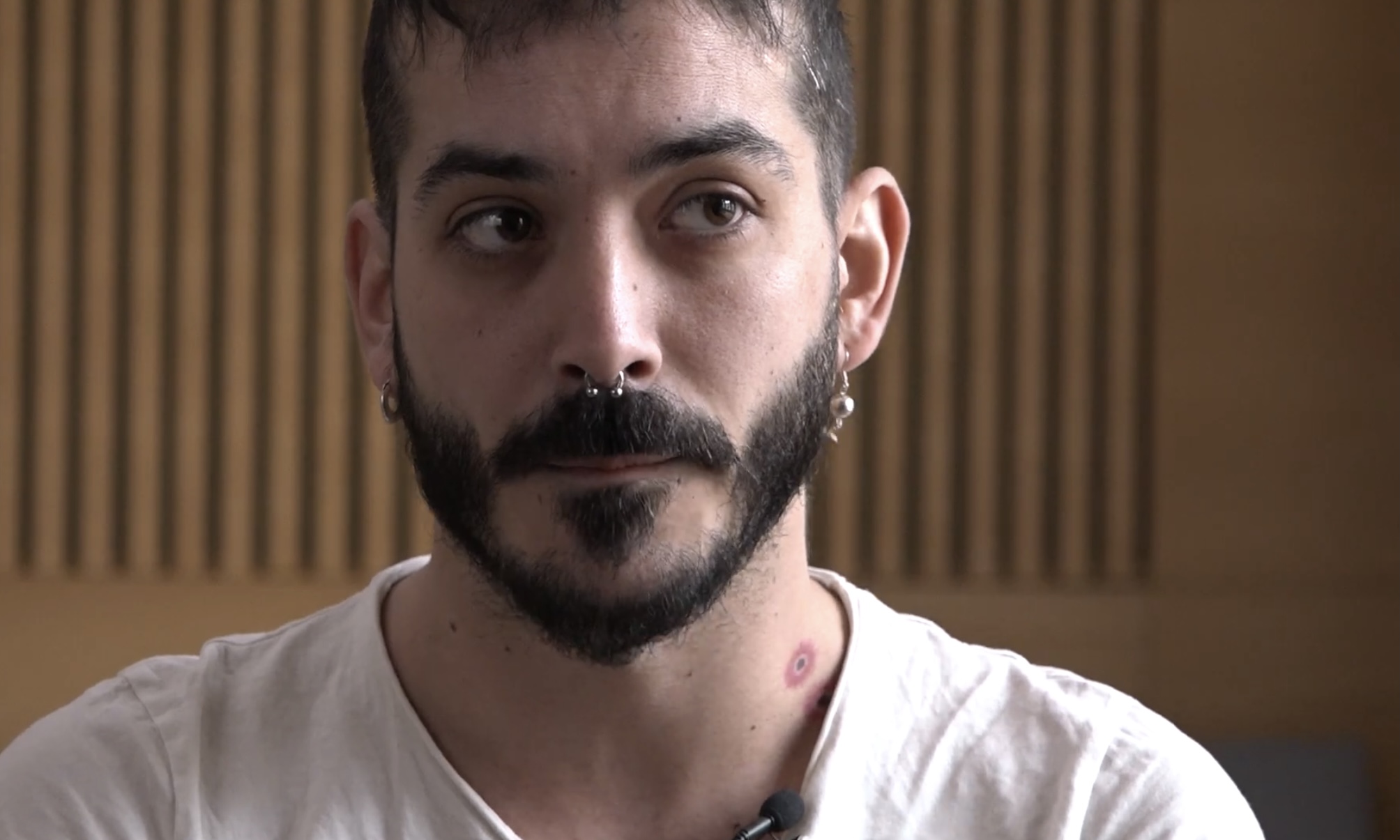


- Looking at the word cloud together. Discussion of written responses: is there anything you would like to add?

*Opinions about the video [these questions are only asked if the topics have not been discussed earlier]:*

What do you think about video in general?

- Has your opinion changed?
- Can the video have an impact on others, why, why not, and how?

*Opinions about the topic of the video [these questions are only asked if the topics have not been discussed earlier]:*

- Why can't people of Romani origin access viewings?
- What could be done about it?
  - What could agents do?
  - What could owners do?

END OF FOCUS GROUP

*Thank you for coming!*

- Do you feel like we left out anything or is there anything you find important to add?
- Information about the gift vouchers: how and when we will deliver them.

**Supplementary Material 2. Codebook**

| Codebook |
| --- |
| NAME OF THE CODE |
| 1. Ideal and non-ideal tenant |
| - 1. Ideal tenant |
| - 1. Non-ideal tenant |
| - 1. Roma as a non-ideal tenant |
| - 1. Attitudes toward Roma |
| - - 1. Feelings |
| - - 1. Prejudices |
| - - 1. Reflection on own attitudes |
| - - 1. Stereotypes |
| - - 1. Othering, boundary creation |
| - - - 1. Practices of identifying Roma |
| - - - 1. References to Roma |
| - 1. Risk (making discrimination necessary) |
| - 1. Trust (necessary to trust the tenant with the valuable asset) |
| 1. Discrimination |
| - 1. Discrimination of Roma |
| - - 1. Attitudes about the discrimination against Roma |
| - - 1. Discrimination of Roma as a social problem |
| - - 1. Experiences |
| - - - 1. Statistical discrimination |
| - - 1. Filtering practices |
| - - - 1. Dialect filter |
| - - - 1. Price filter |
| - - 1. Justification - rationalisation |
| - - - 1. Expectations from others |
| - - - - 1. Expectations from the owner |
| - - - - 1. Expectations of neighbors |
| - - - 1. Professional reputation |
| - - 1. Responsibility |
| - - - 1. Who is responsible for anti-Roma discrimination? |
| - - - 1. Who should solve the problem of anti-Roma discrimination? |
| - - - - 1. Solutions for anti-discrimination |
| - - 1. Risk-reducing strategies |
| - 1. Other groups (discrimination, reference, experience) |
| 1. Group dynamics |
| - 1. General observations about the group |
| - 1. Reaction to other participants |
| - - 1. Conflicting views |
| - - 1. Opinion leader |
| - - 1. Reassurance of agreement |
| - - 1. Submission to opinion leader |
| - 1. Differences between agents and owners |
| - 1. Shifting opinion |
| - - 1. Becoming more prejudiced or discriminatory |
| - - 1. Becoming more tolerant or anti-discriminatory or empathic |
| 1. Prompt |
| - 1. Change after the video |
| - - 1. Change in attitudes |
| - - 1. Change in behavior |
| - - 1. Change in expressions |
| - - 1. Change in intentions for future |
| - 1. Connection to own experiences |
| - 1. Contrast with reality |
| - 1. Emotions |
| - - 1. Empathy |
| - - 1. Feeling sorry |
| - - 1. Frustration |
| - - 1. Hate |
| - - 1. Indifference |
| - - 1. Sadness |
| - - 1. Shame |
| - 1. First reactions |
| - 1. Reaction to video participants |

**Supplementary Material 3.**

**Description of the filmmaking process and the documentary film, used as a prompt**

The film is a collaboration between the Hungarian contingent of the *anonymized* Project research team, *anonymized*, along with resident filmmakers/film educators at *anonymized* University, *anonymized*. Early on in the *anonymized* Project it was determined that the video research products of the investigation could serve as the basis of a film that could raise public awareness among the Hungarian population about the discrimination the Hungarian Roma face in many facets of their everyday lives.

From the start of the process, the research team decided that the creation of the film should take the form of a collaboration with the Roma volunteers, all young adults, to capture these experiences. Instead of an approach that dictated from researchers to subjects what to film and how, a workshop was held in which a collaborative working relationship was established, and volunteers worked alongside the research team to determine what to shoot, and how to shoot it.

It was decided that an approach would be taken that used very small, body-mounted cameras to record these moments of misrecognition, including blatant discrimination. The participants decided what interactions they would record, and how they would approach them. In the end these interactions fall generally into three categories: being denied entry by doormen/bouncers to nightclubs, the process of being singled out and closely monitored by security guards and salespeople in shops, and the process of dealing with real-estate agents in attempting to rent a flat. The participants were then interviewed about these experiences of discrimination on camera, and shared valuable insights, both immediate emotional reactions, as well as keen social analysis.

The filmmakers brought to the film an additional reflexive layer, which involved asking each of the volunteers during their interview, while on camera, to view on a laptop the footage of the interactions they recorded. While watching the completed film, a viewer sees footage of interviews with each group of volunteers, along with the footage they shot of their interactions, and also intercut their reactions while rewatching on a laptop and reacting to that footage. The reliving of the experience brought additional immediacy to their interviews, and to the viewers’ experience of the events taking place in the film. Three advocacy documentaries were produced from the footage, each with a different focus mentioned previously: being denied entry by doormen/bouncers to nightclubs, the process of being singled out and closely monitored by security guards and salespeople in shops, and the process of dealing with real estate agents in renting a flat.

All of the volunteers exposed themselves to risk through their participation in the project, but some more than others. Soon before the release of the videos, one group of volunteers, the subjects of one of the three videos, decided that there could be serious negative repercussions for them in the small city in which they live if the video was made public, and asked that the video not be released. The project team was very supportive of their decision. Therefore, in the end two videos were released and made available on youtube at this *anonymized* link. They were picked up in a story by the Hungarian independent media outlet 444 and were presented in a popular evening magazine by the Hungarian private TV channel, RTL, with 50,000 views. As of the writing of this article, one video has received 33,606 views, the other 6,588 on youtube.

1. Quote from a real-estate agent in the study. [↑](#footnote-ref-1)
2. Questions asked on the Mentimeter platform are presented with a gray background. These questions were answered in writing. Once all participants submitted their responses, responses were presented to the group in a word cloud and were discussed. [↑](#footnote-ref-2)
